# The Secondary Motor Cortex-striatum Circuit Contributes to Suppressing Inappropriate Responses in Perceptual Decision Behavior

**DOI:** 10.1007/s12264-023-01073-2

**Published:** 2023-05-31

**Authors:** Jing Liu, Dechen Liu, Xiaotian Pu, Kexin Zou, Taorong Xie, Yaping Li, Haishan Yao

**Affiliations:** 1 Institute of Neuroscience, State Key Laboratory of Neuroscience, CAS Center for Excellence in Brain Science and Intelligence Technology, Chinese Academy of Sciences, Shanghai, 200031 China; 2 University of Chinese Academy of Sciences, Beijing, 100049 China; 3 Shanghai Center for Brain Science and Brain-Inspired Intelligence Technology, Shanghai, 201210 China

**Keywords:** Secondary motor cortex, Striatum, Visual perceptual decision, Choice signal, Direct and indirect pathway striatal neurons

## Abstract

The secondary motor cortex (M2) encodes choice-related information and plays an important role in cue-guided actions. M2 neurons innervate the dorsal striatum (DS), which also contributes to decision-making behavior, yet how M2 modulates signals in the DS to influence perceptual decision-making is unclear. Using mice performing a visual Go/No-Go task, we showed that inactivating M2 projections to the DS impaired performance by increasing the false alarm (FA) rate to the reward-irrelevant No-Go stimulus. The choice signal of M2 neurons correlated with behavioral performance, and the inactivation of M2 neurons projecting to the DS reduced the choice signal in the DS. By measuring and manipulating the responses of direct or indirect pathway striatal neurons defined by M2 inputs, we found that the indirect pathway neurons exhibited a shorter response latency to the No-Go stimulus, and inactivating their early responses increased the FA rate. These results demonstrate that the M2-to-DS pathway is crucial for suppressing inappropriate responses in perceptual decision behavior.

**Supplementary Information:**

The online version contains supplementary material available at 10.1007/s12264-023-01073-2.

## Introduction

During perceptual decision behavior, humans and other animals process external inputs, evaluate sensory information according to internal state and experience, and produce a motor action [1–3]. Perceptual decision processes involve distributed neural circuits in the brain, particularly sensorimotor cortical regions and the basal ganglia [3]. In rodents, a key region of sensorimotor transformation is the secondary motor cortex (M2), which is a homolog of the primate premotor cortex, supplementary motor area, or frontal eye field [4–6]. M2 receives inputs from sensory and association areas and connects with motor-related regions such as the primary motor cortex, motor thalamus, basal ganglia, and superior colliculus [4, 7, 8]. In both perceptual and value-based decision-making tasks, M2 neurons have been found to exhibit choice-related activity [9–18]. M2 inactivation impairs the performance of perceptual decision-making tasks with or without a delay period [10, 11, 18–23] and causes a deficit in flexible sensorimotor behavior [12, 16]. Thus, the sensorimotor signals in M2 play an important role in perceptual decisions [4, 5].

M2 interacts with other cortical and subcortical regions to guide decision-making behavior [6, 8]. For instance, the projections from M2 to the primary somatosensory cortex (S1) contribute to the late-component activity of layer 5 pyramidal neurons in S1 and enable accurate sensory perception [24]. In a tactile delayed-response task, neurons in the subregion of M2 at the anterior lateral motor cortex (ALM) exhibit persistent preparatory activity critical for motor planning and correct movement [11, 25]. The maintenance of such persistent activity requires an ALM-cerebellar loop [26] as well as reciprocal excitation between the ALM and the thalamus [27]. In a memory-dependent perceptual decision task, a pathway from M2 to the superior colliculus is essential for the maintenance of choice-related information over time [17]. These studies demonstrate that investigation of projection-specific sensorimotor pathways provides important insights into the circuit mechanisms underlying perceptual decision-making.

In addition to projecting to targets in the thalamus and brain stem [17, 25, 27, 28], M2 neurons also send outputs to the dorsal striatum (DS) [7, 29]. Medial M2 projects to the dorsomedial striatum, whereas the ALM projects to the ventrolateral striatum [7, 29, 30]. M2 neurons target striatal medium spiny neurons (MSNs) of both the direct and indirect pathways [31–33], which express D1 and D2 dopamine receptors [34, 35], respectively. Previous studies in primates and rodents have demonstrated that the dorsal striatum plays a causal role in perceptual decisions [36, 37]. Studies in mice are also beginning to examine the role of direct and indirect pathway striatal neurons (D1-MSNs and D2-MSNs) in perceptual decisions. For example, in a tactile whisker-dependent detection task, activation of D1- but not D2-MSNs in the dorsolateral striatum is sufficient for detection performance [38]. In a visual orientation-change detection task, activation of D1-MSNs increases the response bias for visual events in the contralateral visual field, while activation of D2-MSNs increases the response bias for events in both the contralateral and ipsilateral visual fields [39]. It remains unclear, however, how M2 inputs influence decision-related activity in the striatum, and how direct or indirect pathway striatal neurons defined by M2 inputs support perceptual decision behavior.

In this study, we examined the functional role of the M2-DS pathway using a visual Go/No-Go task in mice. We also applied extracellular recordings and fiber photometry to investigate the choice-related signals of M2 and DS neurons. Our experiments revealed that inactivating M2_DS-p_ neurons reduced the choice signal of DS neurons, and the early responses of D2-MSNs defined by M2 inputs played an important role in suppressing FA action. Together, the results reveal the mechanism of the M2-to-DS circuit mediating the suppression of inappropriate responses to a sensory stimulus.

## Materials and Methods

### Animals

All animal procedures were conducted in accordance with the guidelines for the care and use of laboratory animals at the Institute of Neuroscience, Center for Excellence in Brain Science and Intelligence Technology, Chinese Academy of Sciences, and were approved by the Animal Care and Use Committee at the Institute (IACUC No. NA-013-2022). The following strains of mice were used in the experiments: C57BL/6 (SLAC Laboratory Animal Co., Shanghai, China), D1-Cre (Tg(Drd1a-cre)262Gsat/Mmcd) (MMRRC, stock number: 030989-UCD), and D2-Cre (Tg(Drd2-cre)ER44Gsat/Mmcd) (MMRRC, stock number: 032108-UCD). Adult (2–4 months old at the time of surgery) male mice were used for all experiments. The mice were housed under a 12 h:12 h light/dark cycle in the Institute of Neuroscience animal facility (humidity: 40–70%, temperature: 22°C–23°C).

### Surgery

Before surgery, the mice were intraperitoneally injected with a cocktail of fentanyl (0.05 mg/kg), medetomidine (0.5 mg/kg), and midazolam (5 mg/kg), or injected with a mixture of xylazine (40 mg/kg) and zoletil (30 mg/kg). The anesthetized animals were head-fixed in a stereotaxic apparatus. Lidocaine jelly was applied to the incision site. In some experiments, a craniotomy (~ 0.5 mm diameter) was made unilaterally above the central-medial subregion of M2 (AP 1.34 mm, ML 0.75 mm) in the left hemisphere. In other experiments, a craniotomy was made above the ALM (AP 2.46 mm, ML 1.8 mm), the primary tongue/jaw motor area (tjM1) (AP 2 mm, ML 2 mm), or the dorsal striatum (DS, AP 0.26 mm, ML 1.85 mm). The virus (1.5−5×10^12^ viral particles/ml) was injected with a glass pipette (20 μm–40 μm tip diameter).

To inactivate neurons in the central-medial subregion of M2, a total of 250 nL AAV2/8-CaMKIIα-eNpHR3.0-EYFP-WPRE-pA (or AAV2/8-hSyn-eGFP-3Flag-WPRE-SV40p for control mice, which were randomly assigned among cage-mates) was injected at a depth of 800 μm using a syringe pump (Harvard Apparatus, Holliston, USA). To inactivate ALM neurons, a total of 400 nL AAV2/9-CaMKIIα-eNpHR3.0-EYFP-WPRE-hGHpA was injected at a depth of 600 μm into the ALM. To inactivate tjM1 neurons, a total of 250 nL AAV2/9-CaMKIIα-eNpHR3.0-EYFP-WPRE-hGHpA was injected at a depth of 700 μm into the tjM1. To inactivate M2_DS-p_ neurons, 270 nL AAV2-retro-EF1a-mCherry-IRES-Cre-WPRE or AAV2-retro-hSyn-Cre-WPRE-pA was injected at a depth of 2,400 μm into the DS and 500 nL AAV2/8-CAG-DIO-GtACR1-P2A-EGFP (or AAV2/8-EF1a-DIO-EYFP-WPRE for control mice) was injected at a depth of 800 μm into the central-medial subregion of M2. An optical fiber (400 μm diameter, NA 0.37) was placed on the cortical surface above the virus injection site in the central-medial subregion of M2, and 400 μm above the virus injection site in the ALM or tjM1. To inactivate M2 axon terminals in the DS, 250 nL AAV2/8-CaMKIIα-eNpHR3.0-EYFP or AAV2/9-hSyn-eNpHR3.0-EGFP-ER2-WPRE-ployA (or AAV2/8-hSyn-eGFP-3Flag-WPRE-SV40pA as a control) was injected at a depth of 800 μm into the central-medial subregion of M2, and an optical fiber (200 μm diameter, NA 0.37) was inserted to a depth of 1,550 μm into the DS.

For optogenetic tagging of D1-MSNs or D2-MSNs, 400 nL AAV2/9-hSyn-DIO-ChrimsonR-mCherry-WPRE-hGHpA was injected at a depth of 2,100 μm into the DS of D1-Cre or D2-Cre mice.

To inactivate D1-MSNs or D2-MSNs defined by M2 inputs, we used D1-Cre or D2-Cre mice and took advantage of the self-complementary (sc)AAV1 that exhibits anterograde transsynaptic spread [40]. For this experiment, 450 nL scAAV2/1-hSyn-FLEX-Flpo-pA was injected at a depth of 800 μm into the central-medial subregion of M2, 500 nL AAV2/9-hEF1a-fDIO-GTACR1-EGFP-WPRE-pA (or AAV2/9-hSyn-fDIO-somaGCaMP6f-WPRE-hGHpolyA as a control) was injected at a depth of 2,100 μm into the DS, and an optical fiber (200 μm diameter, NA 0.37) was inserted 200 μm above the virus injection site into the DS.

For fiber photometry recordings from M2_DS-p_ neurons, 500 nL AAV2-retro-hSyn-Cre-WPRE-pA was injected at a depth of 2,100 μm into the DS, and 500 nL AAV2/9-EF1a-DIO-GCaMP6f-WPRE-pA was injected at a depth of 800 μm into the central-medial subregion of M2. An optical fiber (400 μm diameter, NA 0.37) was inserted 400 μm above the virus injection site in the central-medial subregion of M2.

For fiber photometry recordings from D1-MSNs or D2-MSNs defined by M2 inputs, we used D1-Cre or D2-Cre mice, in which 450 nL scAAV2/1-hSyn-FLEX-Flpo-pA was injected at a depth of 800 μm into the central-medial subregion of M2, 500 nL AAV2/9-hSyn-fDIO-somaGCaMP6f-WPRE-hGHpolyA was injected at a depth of 2,050 μm into the DS, and an optical fiber (200 or 400 μm diameter, NA 0.37) was inserted 200 μm above the virus injection site in the DS.

A stainless-steel head plate was fixed to the skull using dental cement mixed with 50% carbon powder. In mice used in extracellular recordings, the skull region above M2 or the DS was marked with permanent ink. After the surgery, mice were given Rimadyl *via* drinking water for 3 days. Mice were allowed to recover for at least 10 days prior to behavioral training.

### Behavioral Task

The visual stimuli used in the behavioral task were oriented gratings (90° × 90°, spatial frequency = 0.036 cycles/deg, contrast = 100%), which were presented on a 17" LCD monitor (Dell E1713S, mean luminance 50 cd/m^2^, refresh rate 60 Hz) placed ~12 cm away from the eye contralateral to the recording site or virus injection site. The Go and No-Go stimuli were vertically and horizontally oriented gratings, respectively, and were randomly interleaved. The stimulus presentation period included a waiting period, in which the vertically (horizontally) oriented grating was static, and an answer period, in which the grating drifted rightward (upward).

Before behavioral training, the mice were water-deprived for 2 days. During training, each mouse was head-fixed and rested in an acrylic tube inside a chamber. A lick spout was located ~ 5 mm in front of the tip of the mouse’s nose and 1 mm below the lower lip. Licks were detected as spout contacts by a custom-made electrical lick sensor or the interruption of an infrared beam. Water delivery was controlled by a peristaltic valve (Kamoer, Shanghai, China). An Arduino microcontroller platform was used for stimulus presentation (together with Processing software), lick detection, water delivery, laser stimulation, and data acquisition. The task-related signals and lick signals were sampled at 1000 Hz.

Behavioral training included a habituation phase, a conditioning phase, and a Go/No-Go visual discrimination phase. During the habituation phase (1−2 days), the mouse learned to lick from the lickspout to get a water reward every 1 s. During the conditioning phase (2–3 days), a vertically oriented grating stimulus was presented in each trial. The inter-trial interval included a 6-s blank screen, followed by a flexible interval. During the flexible interval, the screen remained blank, and licking resulted in a 4-s timeout period. Licking in this timeout period triggered another timeout of 4 s unless no lick was detected during the timeout period or an accumulated timeout period >12 s. The grating stimulus was static for 0.5 s (waiting period) and then drifted for 2.5 s (answer period). If a lick was detected during the answer period, the mouse was rewarded with 5 μL of water. During the Go/No-Go visual discrimination phase (10–16 days), the Go and No-Go stimuli (vertically and horizontally oriented gratings, respectively) were randomly interleaved. The stimulus presentation period included a waiting period (0.5 s, grating static) and an answer period (2.5 s, grating drifting), and the inter-trial interval was similar to that in the conditioning phase. In each trial, licking within the waiting period was neither rewarded nor punished, and licks within the answer period were used to determine behavioral performance. For a Go trial, licking within the answer period resulted in 5 μl of water reward (Hit), and no lick was counted as a miss. For a No-Go trial, licking within the answer period was an FA and no lick was a correct rejection (CR). Neither FA nor CR was associated with water reward. Each mouse performed the behavioral task for 1 h in each session.

In a subset of mice, we recorded images of the facial area with a camera (MV-CE018-80UM, Kikvision, Hangzhou, China) at 30 Hz. Infrared LEDs (850 nm) were used to illuminate the face of the mouse.

### Optogenetic Stimulation

The optical activation of NpHR was induced by green light, the activation of GtACR1 was induced by green or blue light, and the activation of ChrimsonR was induced by red light. A green (532 nm), a blue (473 nm), or a red (635 nm) laser (Shanghai Laser & Optics Century Co., Shanghai, China) was connected to an output optical fiber. Laser stimulation was controlled by an Arduino microcontroller.

Laser-OFF and laser-ON blocks (20 trials/block) were interleaved in each session. In laser-ON blocks, laser stimulation was applied during both Go and No-Go trials. In the experiments inactivating M2 neurons (Figs 2, S2, and S3) or M2 axon terminals in the DS (Fig. 2), laser stimulation covered both the waiting period and the first 500 ms of the answer period. In the experiments inactivating M2_DS-p_ neurons (Figs 2, 4, and S5), we applied laser stimulation at different time windows: (1) the waiting period and the first 500 ms of the answer period (Figs 2 and 4), (2) only the waiting period (Fig. S5), or (3) the first 500 ms of the answer period (Fig. S5). In the experiments inactivating D1-MSNs or D2-MSNs defined by M2 inputs (Fig. 6), we applied laser stimulation during the first 300 ms of the waiting period or the first 500 ms of the answer period. The laser was set at a power of 5−8 mW, 4.5−6 mW, and 1−4 mW at the fiber tip for the green, blue, and red laser, respectively.

### Fiber Photometry Recording

Mice that had been trained in the Go/No-Go visual discrimination phase for at least 7 days were used for fiber photometry recordings. A single-channel fiber photometry system was used to record fluorescent Ca^2+^ signals from the M2 or the DS. Light from a 473-nm LED was reflected off a dichroic mirror (MD498, Thorlabs Inc., NJ, USA). The signal was filtered by a bandpass filter (MF525-39, Thorlabs Inc.) and collected in a photomultiplier tube (PMT, R3896, Hamamatsu Photonics, Hamamatsu-city, Japan). The light at the fiber tip was adjusted to 30 μW–40 μW to minimize bleaching. An amplifier converted the PMT output to voltage signals, which were sampled at 200 Hz using a data acquisition card (USB6009, National Instruments, TX, USA) with custom-written programs. Fiber photometry responses were recorded from 1–5 sessions with each mouse. To determine whether the signals recorded in GCaMP6f-expressing mice were caused by movement artifacts, GFP-expressing mice (AAV2/9-CaMKIIα-EGFP-WPRE-hGHpA injected into M2 of C57BL/6 mice or AAV2/5-CAG-FLEX-EGFP-WPRE-pA injected into the DS of D1-Cre/D2-Cre mice) were used as controls [41].

### Optogenetic Tagging

During optogenetic tagging, a red laser (150 trials of 100 ms laser stimulation, with a 5 s inter-trial interval) was applied to the fiber of the optrode. A paired *t* test was used to compare the spike number between the spikes in a 1-s period before laser onset and those in a 6-ms period after laser onset. A unit with *P* <0.01 was considered to be significantly activated by laser stimulation. We further required that the Pearson’s correlation coefficient between the laser-evoked spike waveform and the spontaneous spike waveform was >0.95 [42].

### Extracellular Recording

Mice that had been trained in the Go/No-Go visual discrimination phase for at least 7 days were used for extracellular recordings. Before recordings from behaving mice, the animals were restricted in a circular plastic tube and their head plates were fixed to a holder attached to the stereotaxic apparatus. The animals were anesthetized with isoflurane (1%–2%), and a craniotomy (~ 1 mm diameter) was made above the central-medial subregion of M2 (AP 1.34 mm, ML 0.75 mm) or DS (AP 0.26 mm, ML 1.85 mm). The dura was removed and the craniotomy was covered by a silicone elastomer (Kwik-Cast, WPI, Sarasota, FL, USA). The mice were allowed to recover from the anesthesia in a home cage for at least 1 h. The recordings were made with multi-site silicon probes (A1×32-ploy2-10mm-50s-177-A32, NeuroNexus Technologies, Ann Arbor, MI, USA; ASSY-37-32-1, Diagnostic Biochips, Inc., Maryland, USA). After finishing the recordings, the electrode was retracted. The craniotomy was cleaned with saline and protected with a silicone elastomer. We made 1–5 sessions of recordings from each mouse. For some recordings, the electrode was coated with a fluorescent dye (DiI or DiO, Invitrogen, Eugene, OR, USA) in order to mark the electrode track.

We used a Cerebus 32-channel system (Blackrock Microsystems, Salt Lake City, UT, USA) to amplify and filter the neural signals. Spiking signals were sampled at 30 kHz. We band-pass filtered the signals at 250 Hz–7500 Hz, and used a threshold at 4.0 SD of the background noise to detect the waveforms of spikes. Spikes were sorted offline using commercial software (Offline Sorter V3, Plexon Inc., Dallas, TX, USA) based on cluster analysis of the principal component. If the inter-spike interval was >1 ms and the *P* value for multivariate analysis of variance tests on clusters was <0.05, the spike clusters were considered to be single units.

### Histology

The mice were deeply anesthetized with isoflurane and were perfused with 25 mL saline followed by 25 mL paraformaldehyde (PFA, 4%). The brains were removed and postfixed in 4% PFA overnight at 4℃. After fixation, the brains were transferred to 30% sucrose in phosphate-buffered saline (PBS) until equilibration. The brains were sectioned at 60 μm on a cryostat (Microm, Zeiss, Wallford, Germany). To observe EGFP fluorescence in brain slices from mice in which AAV2/8-CAG-DIO-GtACR1-P2A-EGFP was injected into M2 and AAV2-retro-EF1a-mCherry-IRES-Cre-WPRE into the DS, as well as from mice in which scAAV2/1-hSyn-FLEX-Flpo-pA was injected into M2 and AAV2/9-hEF1a-fDIO-GTACR1-EGFP-WPRE-pA into the DS, the slices were incubated with blocking solution (20% BSA, 0.5% Triton X-100 in PBS) for 2 h at room temperature, followed by incubation with primary antibody (rabbit anti-GFP, 1:1000, Invitrogen, G10362) overnight at 4℃. The slices were rinsed in PBS and incubated with a secondary antibody (donkey anti-rabbit Alexa Fluor 594, 1:1000, Invitrogen, A21207) for 2 h at room temperature. The sections were washed with PBS, mounted onto glass slides, and coverslipped with DAPI Fluoromount-G (SouthernBiotech, 0100-20). Fluorescence images were acquired using VS120 (Olympus, Tokyo, Japan). Images were analyzed with ImageJ (NIH, Bethesda, MD, USA). The atlas schematics for figures in this study are modified from the mouse brain atlas by Franklin and Paxinos [43].

### Data Analysis

Analyses were performed using MatLab (MathWorks Inc., Natick, USA). To quantify behavioral performance, we computed the following:

- Hit rate = number of Hit trials/(number of Hit trials + number of miss trials),
- CR rate = number of CR trials/(number of FA trials + number of CR trials),
- FA rate = number of FA trials/(number of FA trials + number of CR trials),
- Behavioral discriminability (d′) = norminv(Hit rate) – norminv(FA rate), in which norminv is the inverse of the cumulative normal function [44].

For optogenetic experiments, a change in behavioral performance (ΔHit rate, ΔFA rate, or Δd′) was computed as Hit rate (FA rate or d′) in laser-ON trials minus that in laser-OFF trials.

We computed the lick latency in a No-Go trial as the time of the first lick within 1 s after stimulus onset. For those trials in which no lick occurred within 1 s after stimulus onset, the first lick latency was set to 1 s. The lick latency in each session was computed as the median of the first lick latency across trials.

To determine whether an M2 neuron or a DS neuron is responsive to a visual stimulus, we computed the baseline firing rate during the 0.5 s before stimulus onset and the firing rate during the waiting period of stimulus presentation. Those neurons in which the evoked responses during the waiting period were significantly higher than the baseline responses (*P* <0.05, Wilcoxon signed rank test) were included in the analysis. The peri-stimulus time histogram (PSTH) for spiking activity in each trial was constructed with a bin size of 25 ms. For display purposes only, the PSTHs were smoothed by a Gaussian filter using the MatLab function 'smoothdata'. To analyze the choice signal within the waiting period of the No-Go stimulus, we applied receiver operating characteristic (ROC) analysis [45] to the distributions of spike counts on FA and CR trials. To minimize the influence of licking movement, we only analyzed choice signals using those trials in which no lick was detected within the waiting period [46]. The area under the ROC curve (ROC_area_) indicates the accuracy with which an ideal observer can correctly classify whether a given response is recorded under FA or CR conditions. We computed choice preference as 2×(ROC_area_ – 0.5), which ranges from − 1 to 1 [47, 48]. A choice preference with a positive value indicates that the firing rate in the CR trial is lower than that in the FA trial.

To analyze the relationship between the choice preference of M2 (or DS) neurons and CR rate, we grouped neurons according to the CR rate and the bin size of the CR rate was 10%, so that neurons recorded in sessions with a CR rate within [20% 30%] (or [30% 40%], [40% 50%], …, [80% 90%]) were considered as coming from mice with a specific level of behavioral performance. We averaged the choice preference of M2 (or DS) neurons from sessions that belonged to the same bin of CR rate. For instance, the sessions with CR rates of 41% and 46% were included in the bin of 40% < CR rate ≤ 50% and the choice preferences of neurons in this bin of CR rate were averaged. In this analysis, we only included those sessions containing ≥10 FA trials in which no lick occurred within the waiting period. Pearson’s correlation coefficient was computed between the average choice preference and CR rate.

To analyze the fiber photometry responses of M2_DS-p_ neurons or striatal D1-MSNs (D2-MSNs) defined by M2 inputs, the value of fluorescence change (∆F/F) was derived by calculating (F - F_0_)/F_0_, where F_0_ is the baseline fluorescence signal averaged over 1 s before stimulus onset. To compute the responses to the No-Go stimulus in CR (or FA) trials, we averaged the values of ∆F/F during the waiting period. The computation of choice preference and the criteria for session inclusion were similar to those for the analysis of the spiking response.

We analyzed the orofacial movements using FaceMap software (www.github.com/MouseLand/FaceMap) [49]. We computed motion energy as the absolute value of the intensity difference of consecutive images and focused on the first three principal components (PCs) of motion energy [49]. Those trials in which no lick was detected within the waiting period were used for analysis. For each session, we also applied ROC analysis to the motion energy PCs in CR trials and FA trials (Fig. S8), limiting the analysis window to the waiting period.

To compute the latency of ∆F/F for striatal D1-MSNs (D2-MSNs) defined by M2 inputs, we smoothed the response curve (binned at 25 ms resolution) using the 'rloess' method in MatLab, defined ∆F/F during the 0.5 s before stimulus onset as the baseline, and set a threshold as 3 standard deviations above the mean of the 0.5-s baseline. The latency of ∆F/F was calculated as the first time point at which ∆F/F crossed the threshold.

To determine whether the inactivation of M2_DS-p_ neurons significantly affects the firing rates of DS neurons, we compared the firing rates of a DS neuron between laser-OFF and laser-ON conditions in CR (FA, or Hit) trials using the Wilcoxon signed rank test. This yielded three *P* values for CR, FA, and Hit trials, respectively. The firing rate change was considered to be significant if one of the three *P* values was <0.05.

### Statistics

The statistical analysis was performed in MatLab or GraphPad Prism (GraphPad Software Inc., San Diego, USA). The Wilcoxon signed rank test, Wilcoxon rank sum test, one-way ANOVA, or one-way repeated measures ANOVA were used to determine the significance of the effect. Correlation values were computed using Pearson’s correlation. Data are reported as the mean ± SEM.

## Results

### The M2-to-DS Pathway Is Required for Suppressing Inappropriate Responses in the Visual Go/No-Go Task

**Fig. 1:**
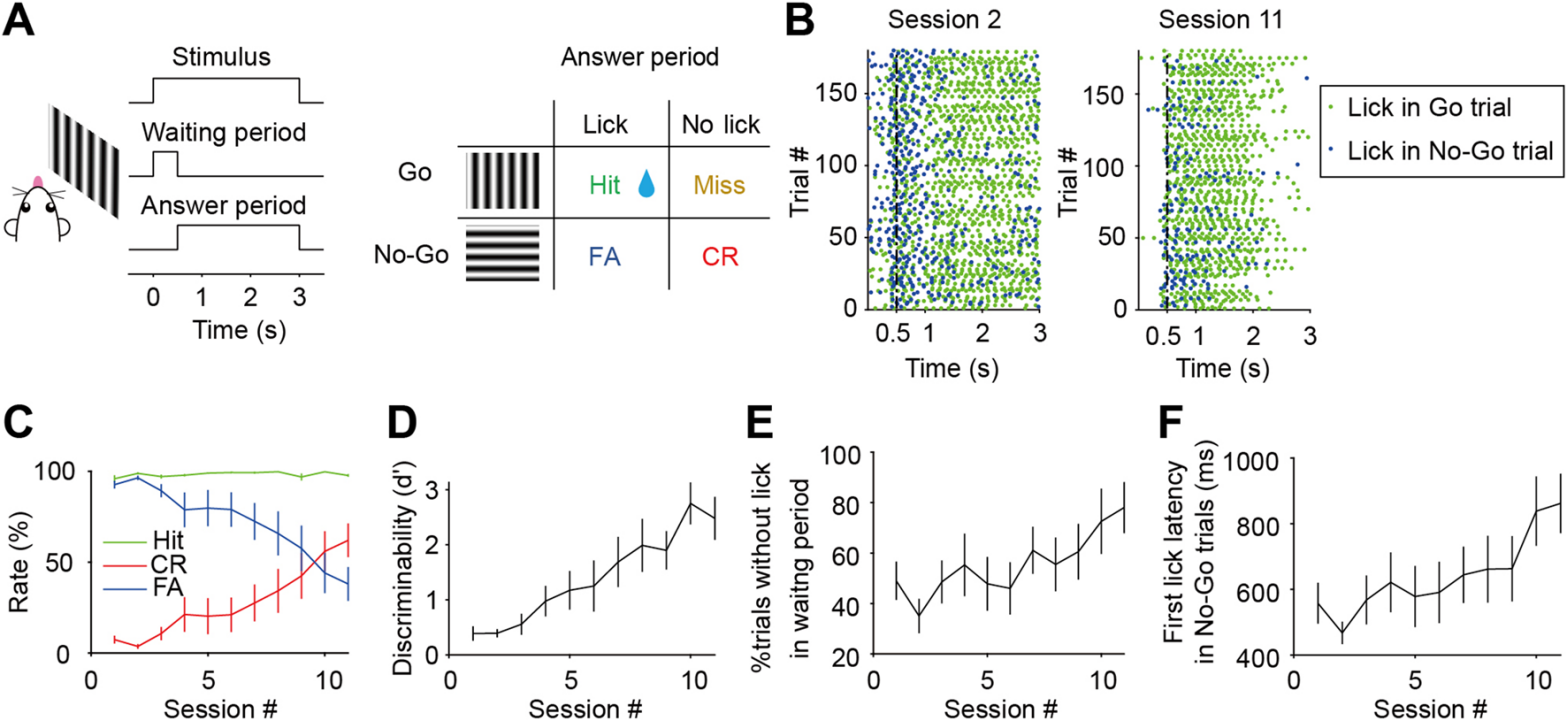
The visual Go/No-Go task in head-fixed mice. **A** Schematic of the task structure in each trial. **B** Example lick rasters in two different sessions. **C** Hit rate, FA rate, and CR rate across training sessions (*P* = 5.33×10^-4^ for CR rate and FA rate, *P* = 0.14 for Hit rate, *n* = 8, one-way repeated measures ANOVA). **D** d′ across training sessions. *P* = 3.64×10^-5^, *n* = 8, one-way repeated measures ANOVA. **E** Percentage of trials in which no lick occurred in the waiting period. *P* = 0.054, *n* = 8, one-way repeated measures ANOVA. **F** Latency of the first lick in No-Go trials across training sessions. *P* = 0.027, *n* = 8, one-way repeated measures ANOVA. Data are represented by the mean ± SEM.

We trained head-fixed mice to perform a Go/No-Go visual discrimination task (Fig. 1A, B) [50]. In each trial, a vertical grating (the Go stimulus) or a horizontal grating (the No-Go stimulus) was presented. The stimulus presentation period included a waiting period and an answer period. For a Go trial, if a lick was detected during the answer period, the mouse was rewarded with water (Hit). In a No-Go trial, licking and no lick within the answer period were considered as FA and correct rejection (CR), respectively, without reward in either case. For both Go and No-Go trials, licking within the waiting period was neither rewarded nor punished. As licking in the Go trials was rewarded and in the No-Go trials was not punished, the mice showed a high Hit rate and high FA rate (low CR rate) in the first session. Over training sessions, the mice learned to withhold licking for the No-Go stimulus (Fig. 1C), with an FA rate of 38% ± 9% (mean ± SEM, *n* = 8) after 11 sessions. The discriminability (d′) between Go and No-Go stimuli improved over training sessions (*P* = 3.64×10^-5^, *n* = 8, one-way repeated measures ANOVA, Fig. 1D). The fraction of No-Go trials in which no licks occurred in the waiting period tended to increase with training (*P* = 0.054, *n* = 8, Fig. 1E), and the latency of the first lick also increased over sessions (*P* = 0.027, *n* = 8, one-way repeated measures ANOVA, Fig. 1F), indicating that the mice gradually understood the temporal structure of the task.

**Fig. 2:**
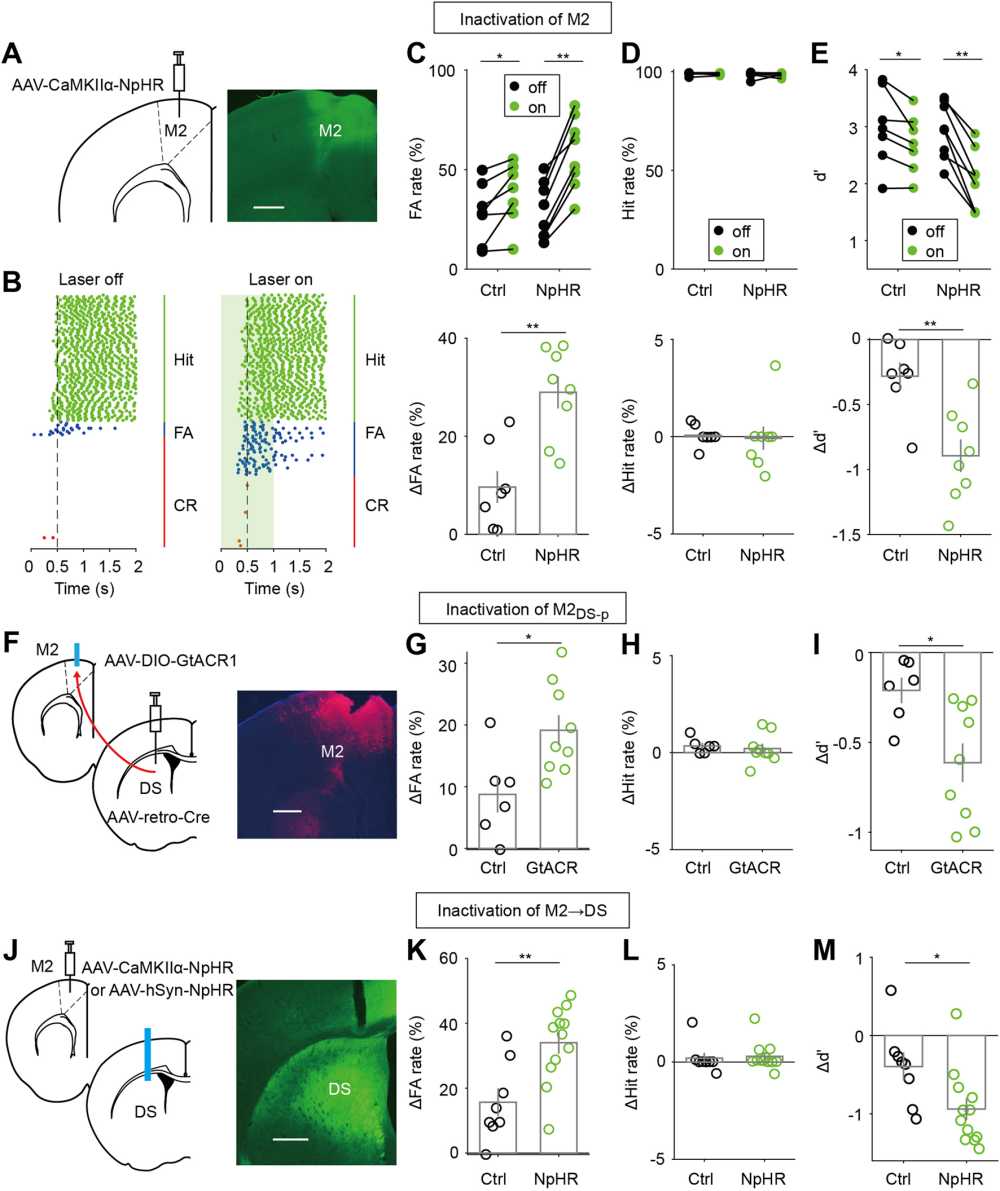
The M2-to-DS pathway is required for suppressing inappropriate responses in the Go/No-Go task. **A** Fluorescence image showing the expression of AAV-CaMKIIα-NpHR-EYFP in M2. **B** Lick rasters of an example mouse in laser-OFF and laser-ON trials. Shading, duration of laser stimulation. **C** Upper, the effect of inactivating M2 on FA rate. *P <0.05, **P <0.01, Wilcoxon signed rank test. Lower, comparison of ∆FA rate between control (*n* = 7) and NpHR (*n* = 8) mice. **P <0.01, Wilcoxon rank sum test. **D** Analysis of Hit rate, as in **C**. **E** Analysis for d′, as in **C**.** F** Schematic of the strategy for inactivating M2_DS-p_ neurons. **G**−**I** Comparison of ∆FA rate (∆Hit rate, or ∆d′) between control (*n* = 6) and GtACR1 (*n* = 9) mice. *P <0.05, Wilcoxon rank sum test. **J** Schematic of the strategy for inactivating M2 axon terminals in the DS. **K**−**M** Comparison of ∆FA rate (∆Hit rate, or ∆d′) between control (*n* = 8) and NpHR (*n* = 12) mice. **P <0.01, *P <0.05, Wilcoxon rank sum test. Data are represented by the mean ± SEM. Scale bars, 500 μm.

To examine whether M2 activity is required for the visual Go/No-Go behavior, we chose to examine the central-medial subregion of M2, which plays an important role in visual conditional motor tasks [51] and cue-guided actions [12]. We unilaterally injected AAV-CaMKIIα-NpHR (or AAV-hSyn-GFP as control) into this subregion of M2 (Fig 2A and S1A). *In vivo*, electrophysiological recordings confirmed that activation of NpHR reduced the firing rates of M2 neurons (Fig. S1B−D). We then trained mice to perform the task by presenting visual stimuli contralateral to the virus injection site. After training, we applied green laser stimulation (532 nm) to M2 during trials of laser-ON blocks that were interleaved with laser-OFF blocks (20 trials/block) (Fig. 2B). We found that laser stimulation significantly increased the FA rate in NpHR mice (*P* = 7.8×10^-3^, *n* = 8, Wilcoxon signed rank test, Fig. 2C). Although laser stimulation also increased the FA rate in the control GFP mice, NpHR mice showed a larger increase in FA rate (*P* = 3.7×10^-3^, *n* = 7 and 8 for control mice and NpHR mice, Wilcoxon rank sum test, Fig. 2C). Laser stimulation did not affect Hit rate in either NpHR mice or control mice (Fig. 2D). We also examined whether laser stimulation affected the licks within the waiting period of Go trials. We found that the laser-induced change in lick rate or early lick fraction was not significantly different between control and NpHR mice (Fig. S2A). Due to the larger increase in FA rate, NpHR mice showed a larger decrease in discriminability (d′) than control mice (*P* = 3.7×10^-3^, *n* = 7 and 8 for control mice and NpHR mice, Wilcoxon rank sum test, Fig. 2E). As we used a block-wise inactivation design, we further examined whether the performance change of NpHR mice occurred within certain blocks in a session. When we analyzed the performance in the first 4 blocks, middle 4 blocks, and last 4 blocks, we found that neither ∆FA rate nor ∆d′ differed significantly across blocks (Fig. S3A, B). We also compared the performance change between the first 10 trials and the last 10 trials in a block and found that ∆ FA rate (or ∆d′) did not differ between the early and later trials (Fig. S3C, D). Together, the results suggest that M2 activity is critical for suppressing inappropriate licking in No-Go trials.

Previous studies have shown that the subregion of M2 in the ALM plays an important role in perceptual decision-making tasks with a delay epoch [11, 25]. In addition to testing the role of the central-medial subregion of M2, we also trained another group of mice to assess the effect of ALM inactivation. We found that inactivation of the central-medial subregion of M2 resulted in a larger increase in FA rate and a larger decrease in d′ than inactivation of the ALM (Fig. S4). This is consistent with the report that the caudal subregion of M2 receives more visual inputs than its rostral subregion [52–54]. As the tjM1 has been found to play a causal role in regulating premature licking in a delayed whisker-detection task [55], we also tested the role of tjM1 in our task. We found that the increase in FA rate induced by the inactivation of tjM1 was significantly smaller than that by the inactivation of the central-medial subregion of M2 (Fig. S4). In subsequent experiments, we thus focused on the central-medial subregion of M2.

To test the causal role of M2_DS-p_ neurons in the Go/No-Go task, we injected AAV-retro-Cre into the DS, and AAV-DIO-GtACR1 (or AAV-DIO-EYFP as a control) into the central-medial subregion of M2 (Fig. 2F). The efficacy of GtACR1-mediated neuronal inhibition was confirmed using *in vivo* recordings (Fig. S1H−J). Compared with the control mice, laser stimulation in GtACR1 mice caused a larger increase in FA rate and a larger decrease in d′ (ΔFA rate: *P* = 0.026; Δd′: *P* = 0.018, *n* = 6 and 9 for control and GtACR1 mice, Wilcoxon rank sum test), without affecting Hit rate (*P* = 0.46, Wilcoxon rank sum test, Fig. 2G−I). We further tested the effect of silencing M2 axon terminals in the DS by injecting AAV-CaMKIIα-NpHR or AAV-hSyn-NpHR into M2 and implanting an optic fiber in the DS (Fig. 2J). For the control mice, AAV-hSyn-eGFP was injected into M2. We found that laser stimulation caused a stronger increase in FA rate and decrease in d′ in NpHR mice than in control mice (ΔFA rate: *P* = 9.7×10^-3^; Δd′: *P* = 0.015, *n* = 8 and 12 for control and NpHR mice, Wilcoxon rank sum test, Fig. 2K−M). In the experiments inactivating M2_DS-p_ neurons or silencing M2 axon terminals in the DS, the ∆FA rate (or ∆d′) did not differ across blocks or between the early and later trials (Fig. S3E−L), and the laser-induced change in waiting-period lick rate or early lick fraction of Go trials were not significantly different between the control and experimental groups (Fig. S2B, C). Thus, the activity of the M2-to-DS pathway is necessary for inhibiting inappropriate responses to No-Go stimuli.

In the above experiments, the laser stimulation lasted 1 s, covering the waiting period and the first 500 ms of the answer period. We further determined the temporal specificity of the effect using those mice in which AAV-retro-Cre was injected into the DS and AAV-DIO-GtACR1 was injected into M2. We found that optogenetic inactivation of M2_DS-p_ neurons during the waiting period caused a significant increase in FA rate (*P* = 0.03) and decrease in d′ (*P* = 0.03, *n* = 6, Wilcoxon signed rank test), whereas inactivation during the first 500 ms of the answer period did not affect behavioral performance (*P* >0.05 for both ΔFA rate and Δd′, *n* = 6, Wilcoxon signed rank test, Fig. S5). Thus, the activity of M2_DS-p_ neurons in the waiting period, during which the mice were presumably forming a decision, is required for suppressing FA response to the No-Go stimulus.

### The Choice Signal of M2 Neurons in Response to the No-Go Stimulus is Correlated with Behavioral Performance

**Fig. 3:**
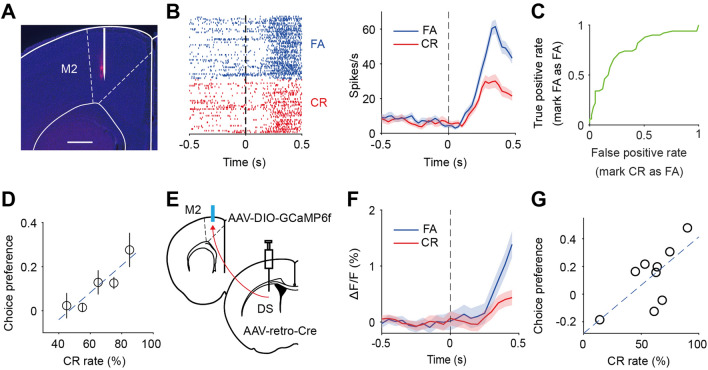
The choice signal in M2 correlates with behavioral performance. **A** Electrode track in M2 marked by DiI. Scale bar, 500 μm. **B** Spike rasters (left) and PSTHs (right) of an example M2 neuron in response to the No-Go stimulus in FA and CR trials. **C** ROC curve for the responses of neurons as in **B**. **D** Choice preference of M2 neurons is positively correlated with CR rate. *r* = 0.92, *P* = 0.026; *n* = 19, 18, 12, 47, and 6 neurons for each level of CR rate, from 8 sessions in 8 mice. Dashed line, the linear fit of the data. **E** Schematic of the strategy for measuring the responses of M2_DS-p_ neurons using fiber photometry. **F** Responses of M2_DS-p_ neurons to the No-Go stimulus in FA and CR trials in an example session. **G** Choice preference of M2_DS-p_ neurons is positively correlated with CR rate. *r* = 0.7, *P* = 0.037; *n* = 9 sessions from 7 mice. Dashed line, the linear fit of the data. Data are represented by the mean ± SEM.

We next recorded from M2 neurons in behaving mice (Fig. 3A). Previous studies have shown that M2 neurons exhibit choice-related signals in cue-guided two-choice tasks [10–17] and visual detection tasks [18]. In the Go/No-Go behavioral task, the responses to the No-Go stimulus in FA *versus* CR trials can be used to estimate choice-related signals [56]. To analyze the choice signal for the No-Go stimulus, we took the responses of M2 neurons in the waiting period and grouped the responses by the choice (FA or CR) (Fig. 3B). We applied ROC analysis (Fig. 3C) to the distribution of spike counts in FA or CR trials, and a choice preference was defined as 2 × (ROC_area_ – 0.5) [48], which ranged from -1 to 1. A positive value of choice preference indicates that the firing rate in the CR trial is lower than that in the FA trial. To reduce the influence of neural responses associated with licking movements, we only analyzed those No-Go trials in which no lick was detected within the waiting period [18, 46]. We found that the choice preference of M2 neurons in response to the No-Go stimulus was positively correlated with the CR rate (*r* = 0.92, *P* = 0.026, Fig. 3D). When we used the responses in the early waiting period (0 – 300 ms) to perform ROC analysis, we found that the choice preference of M2 neurons remained correlated with the behavioral performance (*r* = 0.89, *P* = 0.045, Fig. S6A).

We further used fiber photometry to measure the responses of M2_DS-p_ neurons from mice in which AAV-retro-Cre was injected into the DS and AAV-DIO-GCaMP6f into M2 (Fig. 3E, F). The choice preference of M2_DS-p_ neurons was also positively correlated with the CR rate (*r* = 0.7, *P* = 0.037, Fig. 3G). In control mice expressing GFP, the amplitude of fluorescence signals in FA or CR trials during the waiting period was not significantly different from baseline (Fig. S7A), indicating that the GCaMP6f signals were unlikely to be caused by movement artifacts. These results suggest that, in mice with a stronger ability to suppress licking in the answer period of No-Go trials, M2 neurons show stronger choice-related signals in the waiting period.

To determine whether the choice-related signal was due to movement, we analyzed the orofacial movements in a subset of mice by computing the PCs of motion energy [49] (Fig. S8). We found that the choice preference of motion energy PC in CR trials and FA trials was not significantly correlated with the CR rate (Fig. S8D−F). This suggests that the correlation between the choice preference of M2 neurons and behavioral performance cannot be simply accounted for by changes in orofacial movements with performance.

### The Choice Signal of Striatal Neurons Is Modulated by M2 Activity

**Fig. 4:**
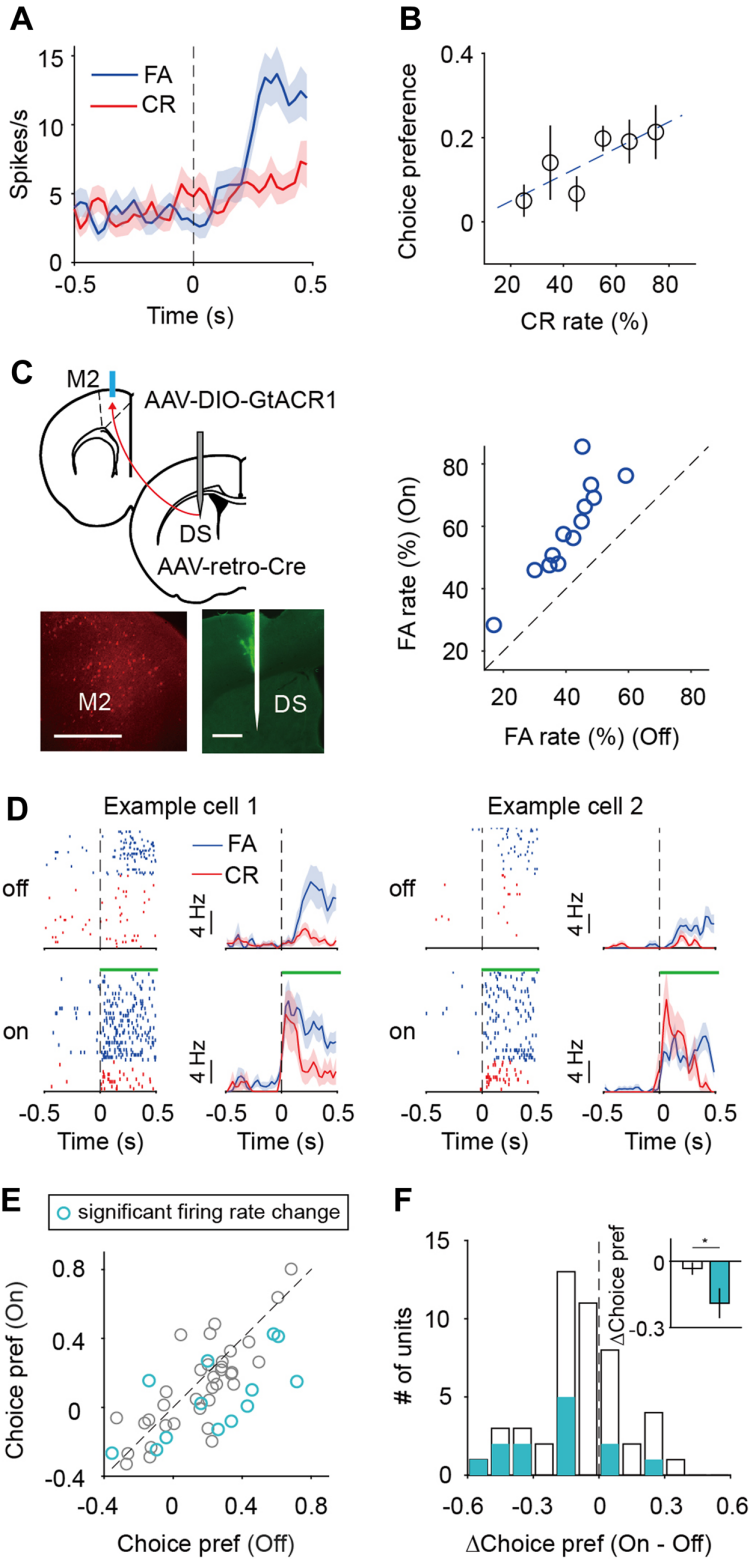
The choice signal in the DS depends on the activity of M2_DS-p_ neurons. **A** PSTHs of an example DS neuron in response to the No-Go stimulus in FA and CR trials. **B** Choice preference of DS neurons is positively correlated with CR rate. *r* = 0.84, *P* = 0.039; *n* = 9, 9, 19, 29, 22, and 10 neurons for each level of CR rate, from 25 sessions in 14 mice. Dashed line, the linear fit of the data. **C** Left, a schematic of the strategy for recording in the DS while inactivating M2_DS-p_ neurons. The electrode track in the DS was marked by DiO. Scale bar, 500 μm. Right, the inactivation of M2_DS-p_ neurons significantly increases the FA rate. *P* = 2.44×10^-4^, *n* = 13 sessions from 10 mice, Wilcoxon signed rank test. **D** Spike rasters and PSTHs of two example DS neurons to the No-Go stimulus in FA and CR trials, with or without inactivation of M2_DS-p_ neurons. Green horizontal lines indicate laser stimulation. **E** Choice preference of DS neurons in laser-OFF *vs* laser-ON trials. *P* = 0.009 and *n* = 48 for all DS neurons, *P* = 0.013 and *n* = 13 for those DS neurons (blue dots) whose firing rates in FA, CR, or Hit trials were significantly affected by inactivation of M2_DS-p_ neurons, Wilcoxon signed-rank test. Dashed line, the diagonal line. **F** Distribution of ΔChoice preference for DS neurons. Blue, DS neurons whose firing rates were significantly affected by the inactivation of M2_DS-p_ neurons. Inset, comparison of ΔChoice preference between DS neurons with an insignificant firing rate change (blue) and those with a non-significant firing rate change (white). *P <0.05, Wilcoxon rank sum test. Data are represented by the mean ± SEM.

Previous studies found choice-related signals in the DS during value-based or perceptual decision-making tasks [57, 58]. We also recorded the part of the DS targeted by M2 projections. The responses of DS neurons to the No-Go stimulus during the waiting period also showed choice-related activity (Fig. 4A, also see Fig. S9 for some D1-MSNs and D2-MSNs identified by optogenetic tagging). Similar to the analysis of M2 neurons, we compared striatal responses to the No-Go stimulus in the waiting period between FA and CR conditions, using those trials in which no licks occurred within the waiting period. We found that the choice preference of DS neurons positively correlated with the CR rate of mice (*r* = 0.84, *P* = 0.039, Fig. 4B), suggesting that the strength of the choice signal in the DS is also related to the task performance in No-Go trials.

We next determined whether the choice signal in the DS is influenced by the activity of M2_DS-p_ neurons. We injected AAV-retro-Cre in DS and AAV-DIO-GtACR1 into M2 (Fig. 4C). After the mice were trained to reach a CR rate >40% (or FA rate <60%), we recorded from DS neurons with or without laser stimulation in M2 (Fig. 4D). In mice used in the electrophysiological recordings, inactivating M2_DS-p_ neurons significantly increased the FA rate (*P* = 2.44×10^-4^, Wilcoxon signed rank test, Fig. 4C). We found that inactivation of M2_DS-p_ neurons caused a significant decrease in choice preference (*P* = 0.009, *n* = 48 neurons, Wilcoxon signed rank test, Fig. 4E, F). As DS neurons receive inputs from multiple cortical areas [7], we assumed that those DS neurons with significant firing rate differences between laser-OFF and laser-ON conditions (see Materials and Methods) may receive direct inputs from M2. In those DS neurons whose firing rate change was significant (blue dots in Figs 4E and S10), inactivation of M2_DS-p_ neurons also caused a significant reduction in choice preference (*P* = 0.013, Wilcoxon signed rank test, *n* = 13 neurons), and this decrease was larger than that of DS neurons with an insignificant firing rate change (*P* = 0.022, Wilcoxon rank sum test, Fig. 4F). When we used the responses in the first 300 ms of the waiting period to apply ROC analysis, we found that the change in choice preference was still larger for DS neurons with a significant firing rate change than those with an insignificant firing rate change (*P* = 0.024, Wilcoxon rank sum test, Fig. S10D, E). These results suggest that the choice signal of DS neurons in response to a No-Go stimulus depends on inputs from M2 neurons.

### The Early Response of Striatal D2-MSNs Defined by M2 Inputs Is Important for Inhibiting FA

**Fig. 5:**
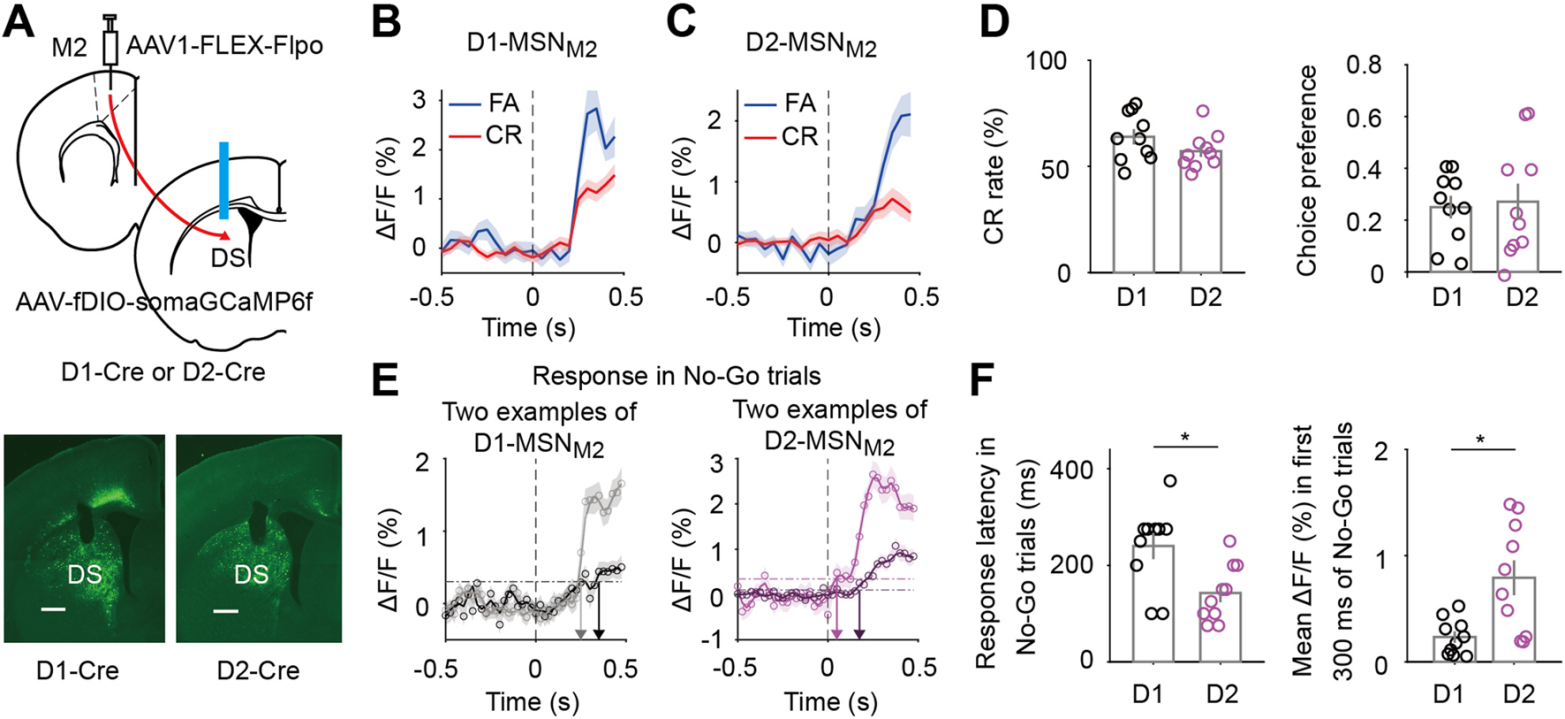
D2-MSN_M2_ shows an earlier response to the No-Go stimulus than D1-MSN_M2_. **A** Schematic of the strategy for measuring the responses of D1-MSNs or D2-MSNs defined by M2 inputs. Scale bar, 500 μm. **B, C** Responses of D1-MSN_M2_ (**B**) and D2-MSN_M2_ (**C**) to the No-Go stimulus in FA and CR trials from example sessions. **D** Left, CR rates in D1-Cre and D2-Cre mice do not significantly differ. *P* = 0.14, Wilcoxon rank sum test. Right, Choice preference does not significantly differ between D1-MSN_M2_ and D2-MSN_M2_. *P* = 0.91, Wilcoxon rank sum test. **E** Illustration of computing latency from the smoothed responses to the No-Go stimulus. Left, two examples of D1-MSN_M2_; the gray curve (latency = 275 ms) corresponds to that in **B**. Right, two examples of D2-MSN_M2_; the dark purple curve (latency = 200 ms) corresponds to that in **C**. Each horizontal dashed line indicates the threshold, which was defined as 3 standard deviations above the mean of the baseline. Arrows point to the latency. **F** Left, response latency in No-Go trials. *P* = 0.011, Wilcoxon rank sum test. Right, mean response within the first 300 ms of the waiting period in No-Go trials. *P* = 0.011, Wilcoxon rank sum test. For **D** and **F**, D1-MSN_M2_: *n* = 10 sessions from 5 mice, and D2-MSN_M2_: *n* = 10 sessions from 5 mice. Data are represented by the mean ± SEM.

Previous studies using rabies-mediated monosynaptic retrograde tracing [59, 60] showed that both D1-MSNs and D2-MSNs in DS are targeted by cortical inputs from M2 [31–33]. To measure the responses of D1-MSNs or D2-MSNs defined by M2 inputs (D1-MSN_M2_ or D2-MSN_M2_), we injected scAAV1-FLEX-Flpo (an anterograde transsynaptic virus [40]) into M2 and AAV-fDIO-somaGCaMP6f into the DS of D1-Cre or D2-Cre mice (Fig. 5A). After the mice were trained in the visual Go/No-Go task and the CR rate was >40%, we made fiber photometry recordings and analyzed the calcium signals in response to the No-Go stimulus within the waiting period (Fig. 5B, C). The behavioral performance did not significantly differ between D1-Cre and D2-Cre mice (Fig. 5D left). The choice preference, computed using responses within the entire waiting period or within the first 300 ms of the waiting period, did not significantly differ between D1-MSN_M2_ and D2-MSN_M2_ (*P* > 0.05, Wilcoxon rank sum test, Figs 5D right and S11A). Interestingly, when we computed the latency of responses in No-Go trials (examples are shown in Fig. 5E), we found that D2-MSN_M2_ had a shorter response latency than D1-MSN_M2_ (*P* = 0.011, Wilcoxon rank sum test, Fig. 5F left), and the mean response in the early waiting period (0–300 ms) was significantly larger in D2-MSN_M2_ than in D1-MSN_M2_ (*P* = 0.011, Wilcoxon rank sum test, Fig. 5F right). Such response differences between D1-MSN_M2_ and D2-MSN_M2_ were also evident in CR trials (Fig. S11B, C).

**Fig. 6:**
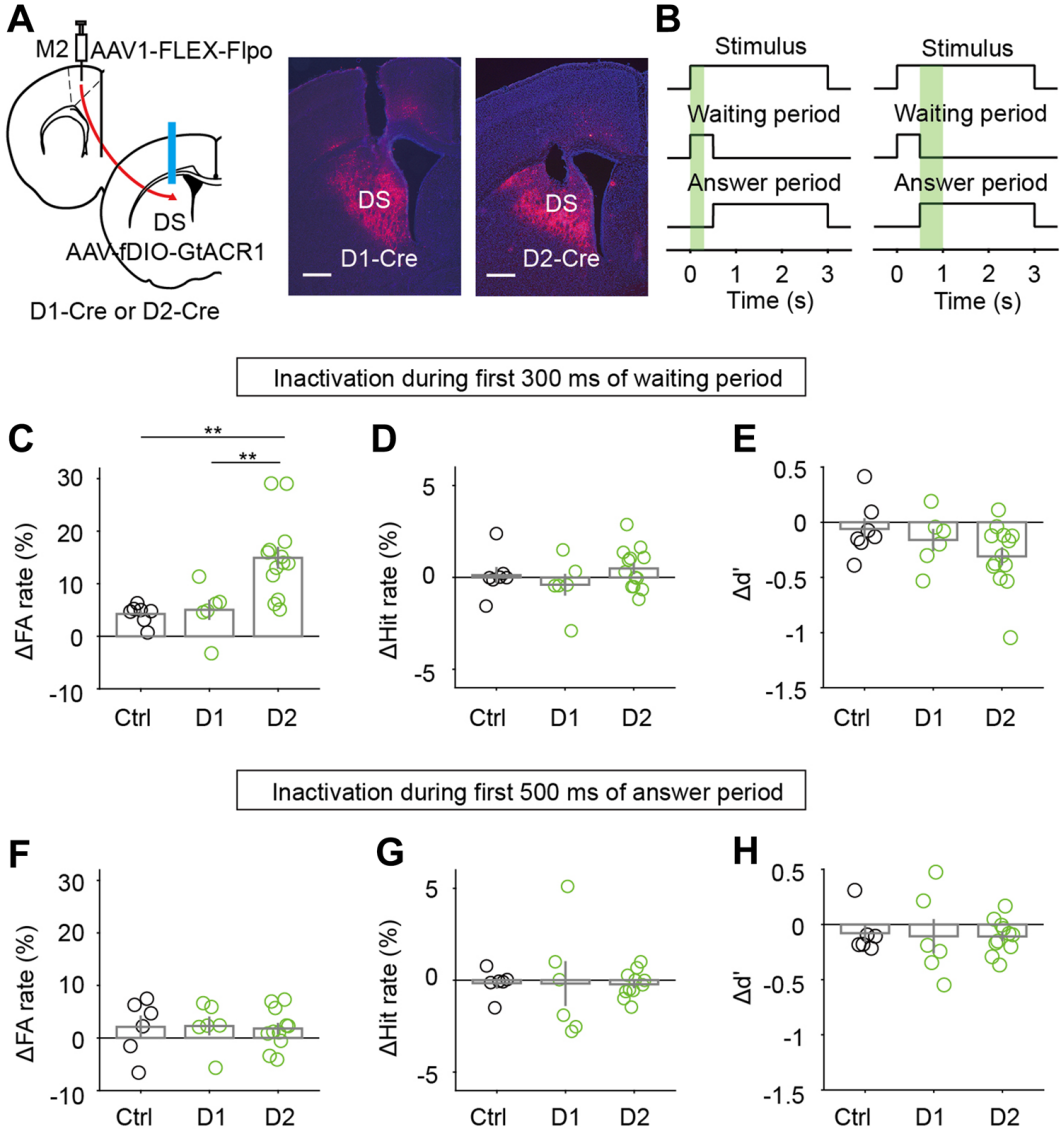
The activity of D2-MSN_M2_ during the early waiting period is important for suppressing false alarms in the Go/No-Go task. **A** Schematic of the strategy for inactivating the responses of D1-MSNs or D2-MSNs defined by M2 inputs. Scale bars, 500 μm. **B** Left, a schematic of laser stimulation during the first 300 ms of the waiting period. Right, a schematic of laser stimulation during the first 500 ms of the answer period. **C**−**E** Comparison of ∆FA rate (∆Hit rate, or ∆d′) among control (*n* = 7), inactivation of D1-MSN_M2_ (*n* = 6), and inactivation of D2-MSN_M2_ (*n* = 13) in experiments in which laser stimulation was applied during the first 300 ms of the waiting period. **P <0.01, one-way ANOVA followed by Dunn and Sidak’s multiple comparisons tests. **F**−**H** Comparison of ∆FA rate (∆Hit rate, or ∆d′) among control (*n* = 6), inactivation of D1-MSN_M2_ (*n* = 6), and inactivation of D2-MSN_M2_ (*n* = 11) in experiments in which laser stimulation was applied during the first 500 ms of the answer period. *P* >0.9, one-way ANOVA. Data are represented by the mean ± SEM.

To test whether the earlier response of D2-MSN_M2_ plays a role in behavioral performance, we trained another group of D1-Cre or D2-Cre mice, in which scAAV1-FLEX-Flpo was unilaterally injected into M2, and AAV-fDIO-GtACR1 injected into the DS (Fig. 6A). After the CR rate reached 40%, we applied laser stimulation through the optic fiber implanted in the DS. Laser-OFF blocks (20 trials/block) were interleaved with laser-ON blocks. Laser stimulation was applied during the first 300 ms of the waiting period so that the responses in the early waiting period were manipulated (Fig. 6B left). Inactivation of D2-MSN_M2_ (*n* = 13) caused a significantly larger increase in FA rate than inactivation of D1-MSN_M2_ (*n* = 6) or laser stimulation in control mice (*n* = 7) (*P* < 0.01, one-way ANOVA followed by Dunn and Sidak’s multiple comparisons tests, Fig. 6C). When we applied laser stimulation during the first 500 ms of the answer period (Fig. 6B right), we found that the change in FA rate (Hit rate, or d′) did not significantly differ among the three groups of mice (*P* > 0.9, one-way ANOVA, Fig. 6F−H). This indicates that the effect of inhibiting D2-MSN_M2_ in the early waiting period is not due to a change in licking movement. Together, the results suggest that the activity of D2-MSN_M2_ during the early waiting period plays an important role in suppressing the FA response to the No-Go stimulus.

## Discussion

In this study, by combining Go/No-Go tasks and pathway-specific optogenetic manipulation, along with electrophysiological and fiber photometry recordings, we examined the role of the M2-DS circuit in visual perceptual decision-making. We found that M2_DS-p_ neurons and the projections from M2 to DS played a causal role in suppressing inappropriate licking for the No-Go stimulus. Both M2 and DS neurons exhibited choice-related signals in response to the No-Go stimulus, and the inactivation of M2_DS-p_ neurons reduced the choice signal in the DS. Compared to striatal D1-MSN_M2_, D2-MSN_M2_ showed an earlier response to the No-Go stimulus. Furthermore, inhibiting D2-MSN_M2_ during the early waiting period led to a larger increase in FA rate than the inactivation of D1-MSN_M2_. These results demonstrate that specific cell types in the M2-DS circuit are essential for withholding a response to a reward-irrelevant stimulus.

Previous studies have demonstrated that M2 plays an important role in perceptual decisions [4–6, 8]. It has been shown that M2 is involved in perceptual behaviors especially when the animals are engaged [22] or the task is demanding [12, 16]. In the Go/No-Go task, while a high Hit rate in response to the Go stimulus occurred in the first session, the withholding response to the No-Go stimulus was more difficult and took more sessions to develop (Fig. 1C). We found that M2 inhibition increased the FA rate but did not affect the Hit rate or lick rate in Go trials during the waiting period, which is consistent with the notion that the function of M2 depends on behavioral engagement [6] and a recent study showing that inactivation of M2 does not affect the performance of Hit trials in a visual detection task [61]. On the other hand, M2 is a large area spanning the frontal cortex along the caudal-rostral axis [43]. The virus injected in the central-medial subregion of M2 spread along the caudal-rostral axis, approaching AP 2 mm and AP 1 mm (Fig. S1A). In the experiment of silencing M2 fibers in the DS, we likely manipulated the fibers of neurons not only from M2 at AP 1.34 mm, ML 0.75 mm but also from the primary whisker motor cortex (AP 1 mm, ML 1 mm) and secondary whisker motor cortex (AP 2 mm, ML 1 mm), which play an important role in the performance of a Go/No-Go delayed whisker-detection task [55]. A previous study showed that the ALM but not medial M2 is involved in a whisker-based object localization task with a delay epoch [62]. Here, we found that the inactivation of the central-medial subregion of M2 caused a larger increase in FA rate than the inactivation of the ALM. This is consistent with the difference in their connections with the visual cortex: the caudal and medial subregions of M2 have stronger connections with primary and higher visual cortices than the rostral-lateral subregion of M2 [52–54].

M2 neurons project to multiple brain regions associated with sensory processing, decision-making, or motor control [4, 6, 8, 53]. Recent studies have used sensorimotor and perceptual decision-making behaviors to examine the role of specific M2 circuits, including the circuits with sensory cortices [24, 63–65], thalamus [27], cerebellum [26], superior colliculus [17], and basal ganglia [66, 67]. As the main input nucleus of the basal ganglia, the striatum receives prominent projections from M2 as well as from other cortical areas [7, 68]. Previous studies on the M2-striatum circuit have demonstrated its contribution to a variety of behaviors, including compulsive behaviors in a mouse model of obsessive-compulsive disorder [69], motor control in mouse models of Parkinson’s disease and Huntington’s disease [70, 71], performance in a serial order task [72], bilaterally coordinated movements in a bimanual coordination task [73], and using action-related experiential information to guide behavior [74]. Although much evidence implicates M2 [10, 12, 16, 22, 75] and the striatum [37, 76, 77] in perceptual decision-making in addition to motor control, few studies have investigated the role of the M2-striatum circuit in perceptual decision-making. Our study adds to the literature by providing evidence that the M2 projections to the DS regulate the choice-related signal in the DS to influence visual perceptual decisions.

Studies on decision-making have shown that M2 neurons exhibit choice-related activity during decision formation or motor planning [9–18, 75, 78], and projection-specific M2 neurons send choice-related information to downstream targets [17, 28]. In the Go/No-Go task, the choice signal is estimated by comparing the responses to the same stimulus between correct and error trials [18, 56]. In our study, we analyzed the choice signal to the No-Go stimulus by computing choice preference using FA and CR trials, and we used those trials in which no lick was detected during the waiting period to minimize the influence of movement [46]. In the population of M2 neurons recorded extracellularly, we found a positive correlation between the choice preference and the CR rate, suggesting that the degree of response difference between FA and CR trials in the waiting period reflects how likely the mice can withhold a response during the answer period. Such a performance-dependent choice signal was also present in M2_DS-p_ neurons, suggesting that the signal was sent downstream to the DS. When M2_DS-p_ neurons were inactivated, the choice preference of DS neurons was reduced, demonstrating that the choice signal in M2 indeed flows to the DS. Consistent with the covariation between choice preference and CR rate, the inactivation of M2_DS-p_ neurons during the waiting period impaired behavioral performance, suggesting that the choice signal is critical for suppressing the FA response. The performance impairment was unlikely to be explained by an effect of inactivation on licking movement because inactivation of M2_DS-p_ neurons during the answer period did not affect performance. Inactivation of M2_DS-p_ neurons tended to increase the firing rates of DS neurons in CR trials (Fig. S10A). As both medium spiny neurons and inhibitory interneurons in the DS receive inputs from M2 [31–33, 79] and the M2-related motor cortex can influence DS activity *via* long-range GABAergic projections [80], the mechanism underlying the modulation of DS firing rates by M2_DS-p_ neurons remains to be investigated.

Similar to M2, the striatum is involved in decision-making as well as motor control [81, 82]. It has been shown that striatal neurons in the direct and indirect pathways are both activated during voluntary movement [83–89], yet they play different roles in regulating movement [90]. Recent studies have also shown that D1-MSNs and D2-MSNs represent decision variables differently. For instance, as the value increased in a probabilistic Pavlovian conditioning task, D1-MSNs and D2-MSNs tended to increase and decrease activity, respectively [91]. In an operant conditioning task, D1-MSNs increased activity during reward delivery, whereas D2-MSNs were more active during no-reward than reward [92]. In a whisker-based detection task, D1- but not D2-MSNs exhibited an early sensory response, and the activity of D1- but not D2-MSNs in the dorsolateral striatum contributed to task performance [38]. Because optogenetic stimulation may cause synchronous activation [93], it is important to use a loss-of-function approach to determine how the endogenous activity of striatal neurons in the direct and indirect pathways contributes to a perceptual decision. As the striatum is innervated by a large number of cortical areas [7], it is also important to examine D1-MSNs and D2-MSNs defined by specific inputs, in terms of activity patterns and contribution to decision-making performance. A recent study revealed that striatal neurons in the direct and indirect pathways defined by ALM inputs oppositely influence choice in a tactile-based decision task with a delay epoch [94]. In our study, we found that D1-MSN_M2_ and D2-MSN_M2_ exhibited similar choice preference during the early waiting period in well-trained mice; however, the response latency to the No-Go stimulus was shorter in D2-MSN_M2_ than in D1-MSN_M2_. As both D1-MSN_M2_ and D2-MSN_M2_ likely receive inputs from multiple regions of the cortex and thalamus, at this stage we could not determine the circuit mechanism underlying the latency difference. Nevertheless, the latency difference is consistent with previous reports that the MSNs in the indirect pathway tend to fire before MSNs in the direct pathway in goal-directed behavior [95] and the two types of MSNs differ in relative timing during movements [89, 96]. We further found that inactivating the early responses of D2-MSN_M2_ resulted in a larger increase in FA rate than inactivation of D1-MSN_M2_, consistent with a recent study showing that suppression of action preferentially engages striatal neurons in the indirect pathway [97]_._ Inactivating D2-MSN_M2_ during the answer period did not impair the behavioral performance, in line with a previous report that striatum inactivation during the stimulus presentation period but not during the post-choice period impairs the performance of decision-making behavior [37]. In future studies, it will be of interest to trace the downstream targets of the M2-DS pathway [98–100] and further investigate how the temporally distinct responses of D1-MSN_M2_ and D2-MSN_M2_ regulate task-related signals in other brain regions.

### Supplementary Information

Below is the link to the electronic supplementary material.

Supplementary file1 (PDF 1413 KB)
