# Supplementary material for: The Secondary Motor Cortex-striatum Circuit Contributes to Suppressing Inappropriate Responses in Perceptual Decision Behavior
Source: Neurosci Bull. 2023 May 31;39(10):1544–60. doi: 10.1007/s12264-023-01073-2 (PMC10533474; doi:10.1007/s12264-023-01073-2)
Supplement: Supplementary file 1 — Supplementary file1 (PDF 1413 KB) [file 12264_2023_1073_MOESM1_ESM.pdf]

Supplementary Figures and Figure Legends

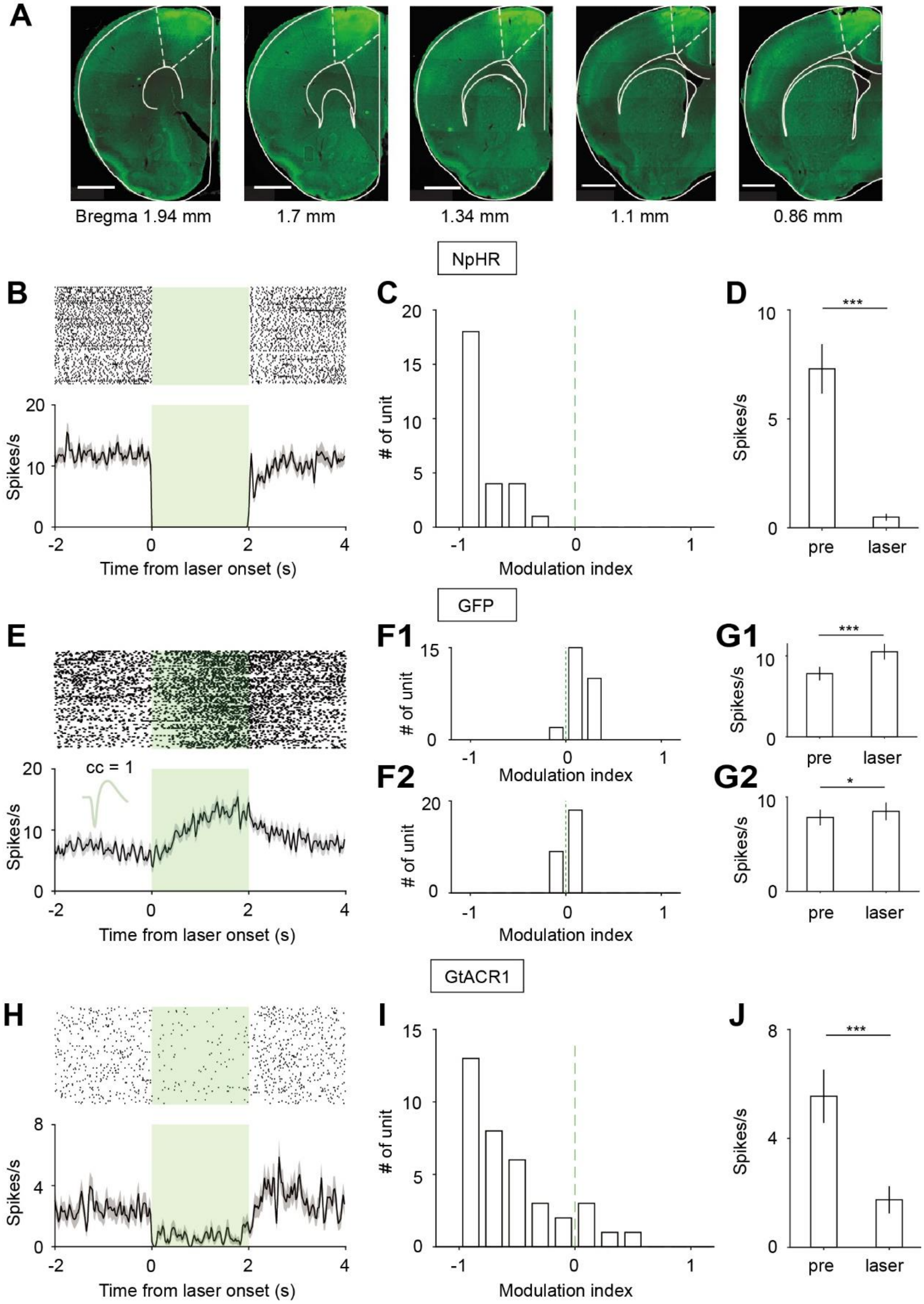

Fig. S1 Electrophysiological recordings to test the efficacy of NpHR- or GtACR1-mediated inhibition. **A** Representative fluorescence images showing the expression of AAV2/8-CaMKIIα-eNpHR3.0-EYFP-WPRE-pA, which was injected in the part of M2 at (AP 1.34 mm, ML 0.75 mm). Scale bars, 1 mm. **B** Spike rasters and PSTH of an example M2 neuron from a mouse in which AAV2/8-CaMKIIα-eNpHR3.0-EYFP-WPRE-pA was injected into M2. Green shading, duration of laser stimulation. **C** Distribution of modulation index, defined as $(R_{laser} - R_{pre})/(R_{laser} + R_{pre})$, in which R_{laser} and R_{pre} are firing rates within a 2-s period during and before laser stimulation, respectively. $P = 5.61\times10^{-6}$, $n = 27$ M2 neurons from NpHR expressing mouse. **D** Firing rates before and during laser stimulation. *** $P < 0.001$, $n = 27$ M2 neurons from NpHR expressing mouse. **E** Spike rasters and PSTH of an example M2 neuron expressing GFP (from a mouse in which the control virus AAV2/9-CaMKIIα-EGFP-WPRE-hGHpA was injected into M2). Green shading, duration of laser stimulation. Inset, spike waveforms before and during laser stimulation. The correlation coefficient (cc) between the two waveforms was 1. **F1** Distribution of modulation index for responses within a 2-s period during and before laser stimulation. $P < 0.001$, $n = 27$ M2 neurons from GFP-expressing mouse. **G1** Firing rates within a 2-s period before and during laser stimulation. *** $P < 0.001$, $n = 27$ M2 neurons from GFP-expressing mouse. **F2** Distribution of modulation index for responses between 2-s before laser and the first 1-s during laser stimulation. $P = 0.14$. **G2** Firing rates within a 2-s period before laser and in the first 1-s period during laser stimulation. * $P = 0.037$. **H** Spike rasters and PSTH of an example M2 neuron from a mouse in which AAV2-retro-hSyn-Cre-WPRE-pA and AAV2/8-CAG-DIO-GtACR1-P2A-EGFP were injected into M2. Green shading, duration of laser stimulation. **I** Distribution of modulation index. $P = 9.08\times10^{-7}$, $n = 37$ M2 neurons from GtACR1-expressing mouse. **J** Firing rates before and during laser stimulation. *** $P < 0.001$, $n = 37$ M2 neurons from GtACR1-expressing mouse. For **C**, **D**, **F1-G2**, **I,** and **J**, Wilcoxon signed-rank test. Data are represented by the mean ± SEM.

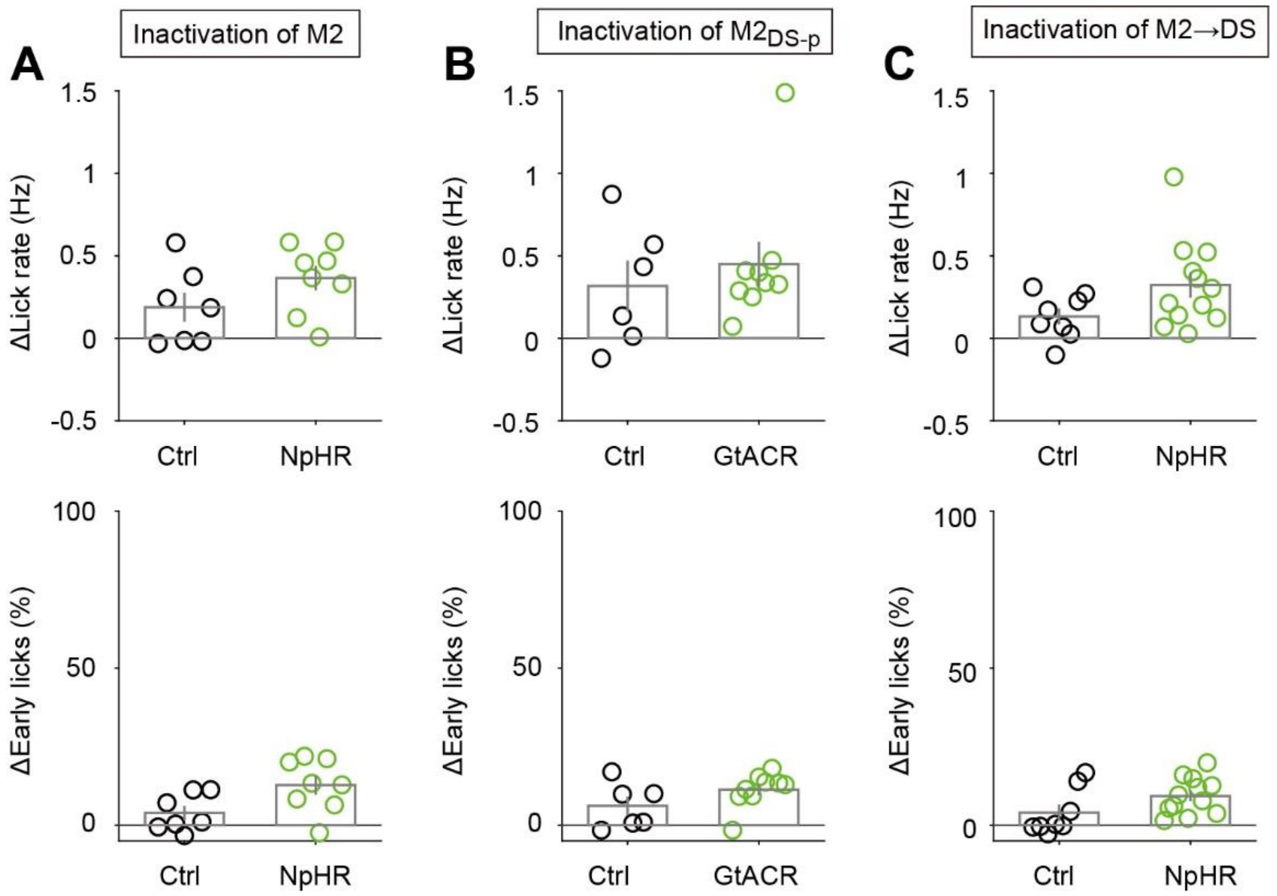

Fig. S2 The change of licks during the waiting period in Go trials. **A** ΔLick rate (lick rate in laser-on trials minus that in laser-off trials) (upper panel) and ΔEarly lick fraction (early lick fraction in laser-on trials minus that in laser-off trials) (lower panel) did not significantly differ between control mice (n = 7) and mice with M2 inactivation (n = 8). **B** ΔLick rate (upper panel) or ΔEarly lick fraction (lower panel) did not significantly differ between control mice (n = 6) and mice with inactivation of $M2_{DS\text{-}p}$ neurons (n = 9). **C** ΔLick rate (upper panel) or ΔEarly lick fraction (lower panel) did not significantly differ between control mice (n = 8) and mice with inactivation of M2 fibers in DS (n = 12). $P > 0.05$, Wilcoxon rank sum test. Data are represented by the mean ± SEM.

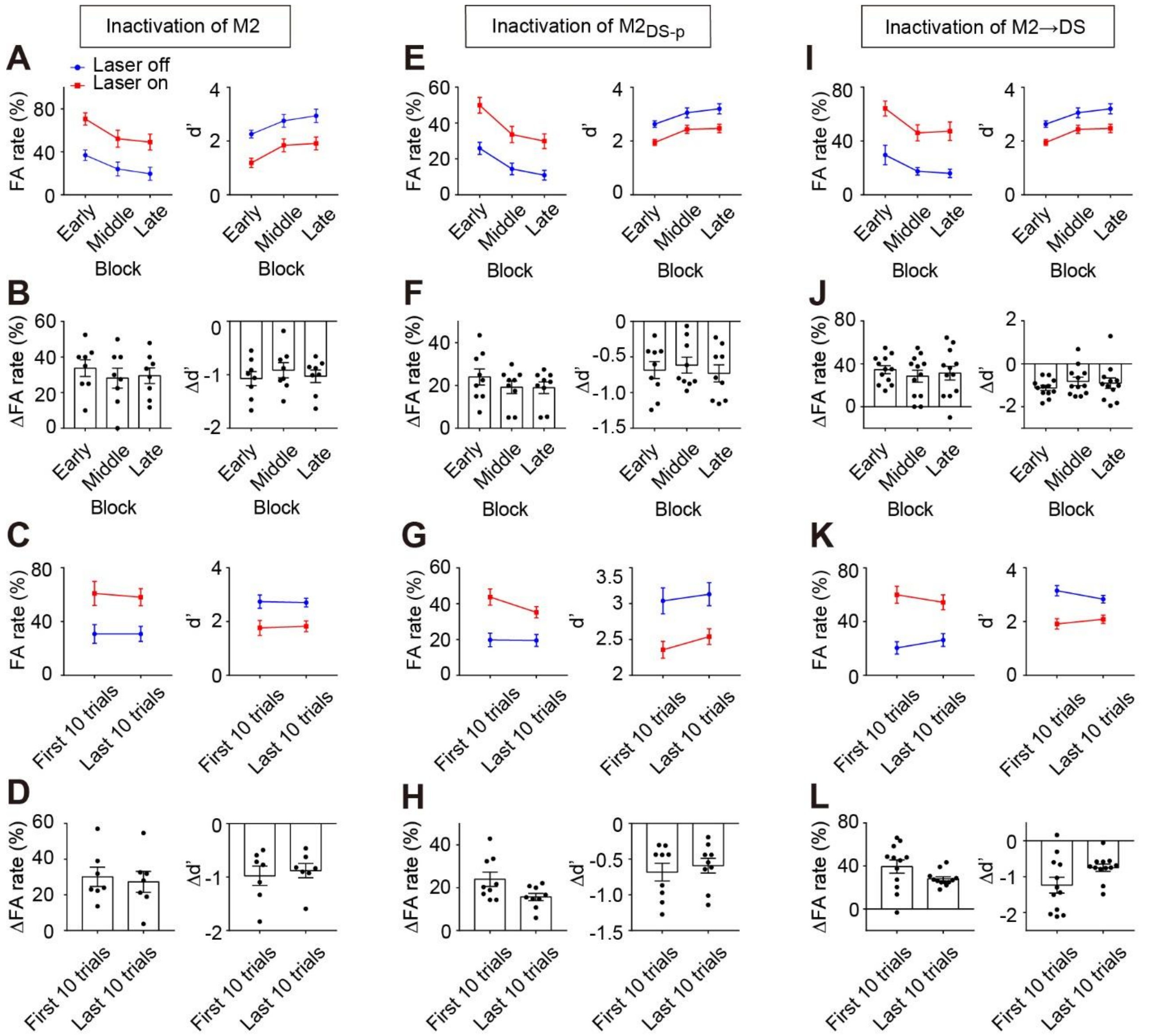

Fig. S3 Comparison of the inactivation effects among different blocks in a session or between early and later trials in a block. **A** FA rate (left) and d′ (right) in different blocks (early block: the first 4 blocks; middle block, the middle 4 blocks; late block, the last 4 blocks) for mice of M2 inactivation. Blue, laser off; red, laser on. **B** ΔFA rate (left) and Δd′ (right) in different blocks for mice of M2 inactivation. $P > 0.3$, $n = 8$, one-way repeated measures ANOVA. **C** FA rate (left) and d′ (right) in the first 10 trials and the last 10 trials in a block, for mice of M2 inactivation. **D** ΔFA rate (left) and Δd′ (right) in the first 10 trials and the last 10 trials in a block, for mice of M2 inactivation. $P > 0.5$, $n = 8$, Wilcoxon signed-rank test. **E** FA rate (left) and d′ (right) in different blocks for mice of $M2_{DS\text{-}p}$ inactivation. **F** ΔFA rate (left) and Δd′ (right) in different blocks for mice of $M2_{DS\text{-}p}$ inactivation. $P > 0.3$, $n = 9$, one-way repeated measures ANOVA. **G** FA rate (left) and d′ (right) in the first 10 trials and

the last 10 trials in a block, for mice of $M2_{DS\text{-}p}$ inactivation. **H** ΔFA rate (left) and Δd′ (right) in the first 10 trials and the last 10 trials in a block, for mice of $M2_{DS\text{-}p}$ inactivation. $P > 0.07$, $n = 9$, Wilcoxon signed-rank test. **I** FA rate (left) and d′ (right) in different blocks for mice with inactivation of M2 fibers in the DS. **J** ΔFA rate (left) and Δd′ (right) in different blocks for mice with inactivation of M2 fibers in the DS. $P > 0.2$, $n = 12$, one-way repeated measures ANOVA. **K** FA rate (left) and d′ (right) in the first 10 trials and the last 10 trials in a block, for mice with inactivation of M2 fibers in the DS. **L** ΔFA rate (left) and Δd′ (right) in the first 10 trials and the last 10 trials in a block, for mice with inactivation of M2 fibers in the DS. $P > 0.07$, $n = 12$, Wilcoxon signed-rank test. Data are represented by the mean ± SEM.

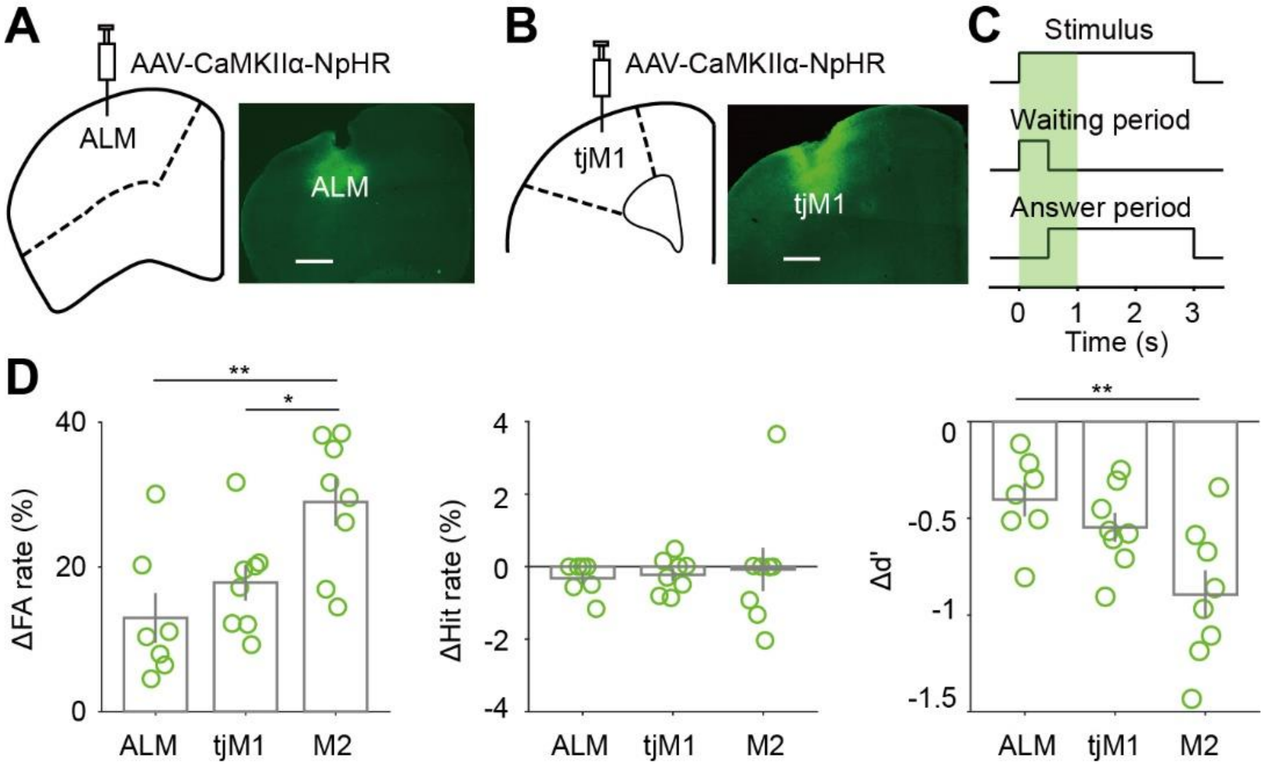

Fig. S4 Comparison of the inactivation effects among three cortical regions. **A** Fluorescence image showing the expression of AAV-CaMKIIα-NpHR in the ALM. Scale bar, 500 μm. **B** Fluorescence image showing the expression of AAV-CaMKIIα-NpHR in the tjM1. Scale bar, 500 μm. **C** Schematic of the duration of laser stimulation. **D** ΔFA rate (ΔHit rate, or Δd') for inactivation of the ALM (n = 7), inactivation of the tjM1 (n = 8), and inactivation of the central-medial subregion of M2 (n = 8, same as those in **Fig. 2**). ** $P < 0.01$, * $P < 0.05$, one-way ANOVA followed by Dunn and Sidak's multiple comparisons tests. Data are represented by the mean ± SEM.

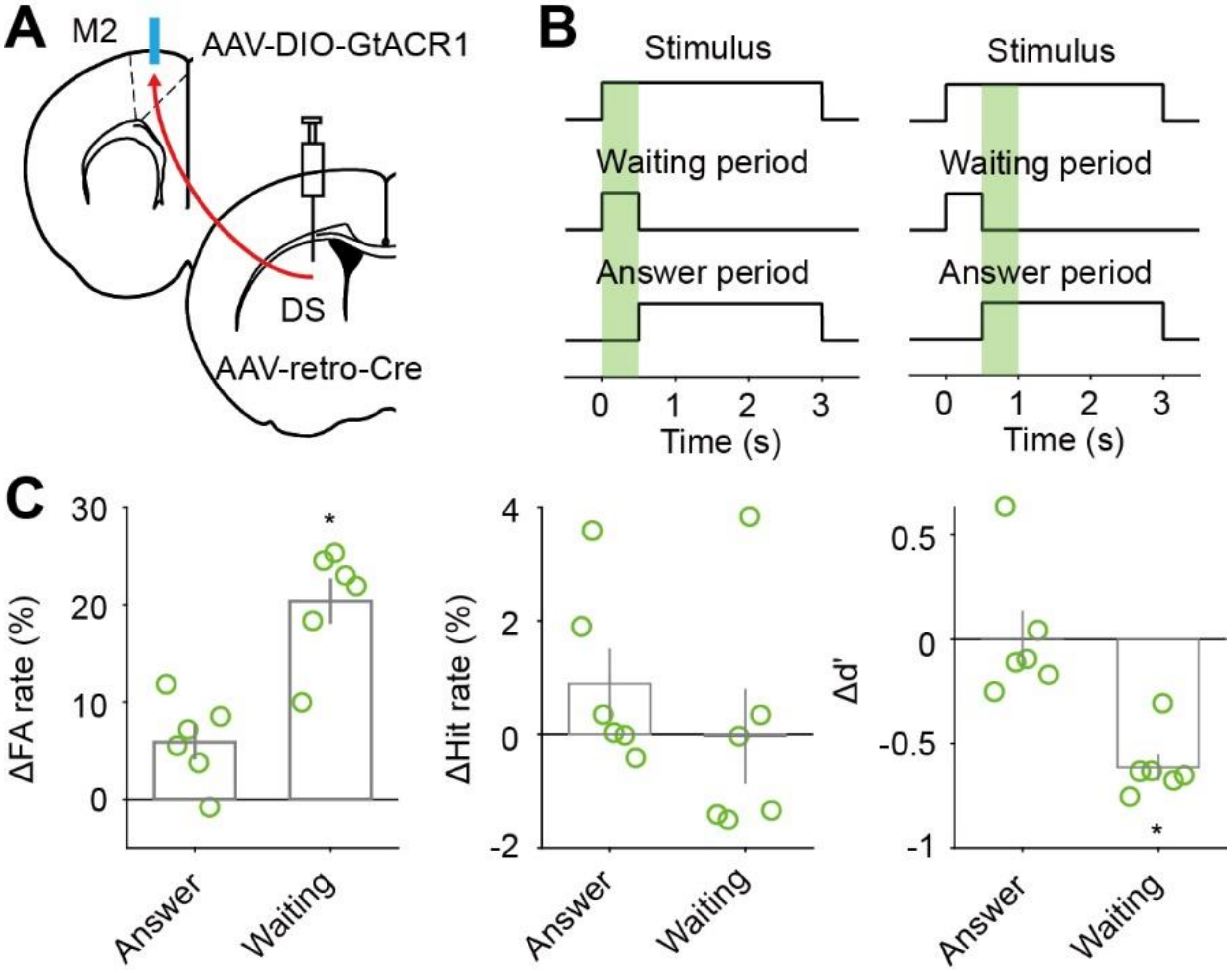

Fig. S5 Temporal specificity of the effect of inactivating M2$_{DS\text{-}p}$ neurons. **A** Schematic of the strategy for inactivating M2$_{DS\text{-}p}$ neurons. **B** Schematic of laser stimulation during the waiting period (left) or the first 500 ms of the answer period (right). **C** ΔFA rate (ΔHit rate, or Δd′) for waiting period manipulation and answer period manipulation. * $P < 0.05$, $n = 6$, Wilcoxon signed-rank test. Data are represented by the mean ± SEM.

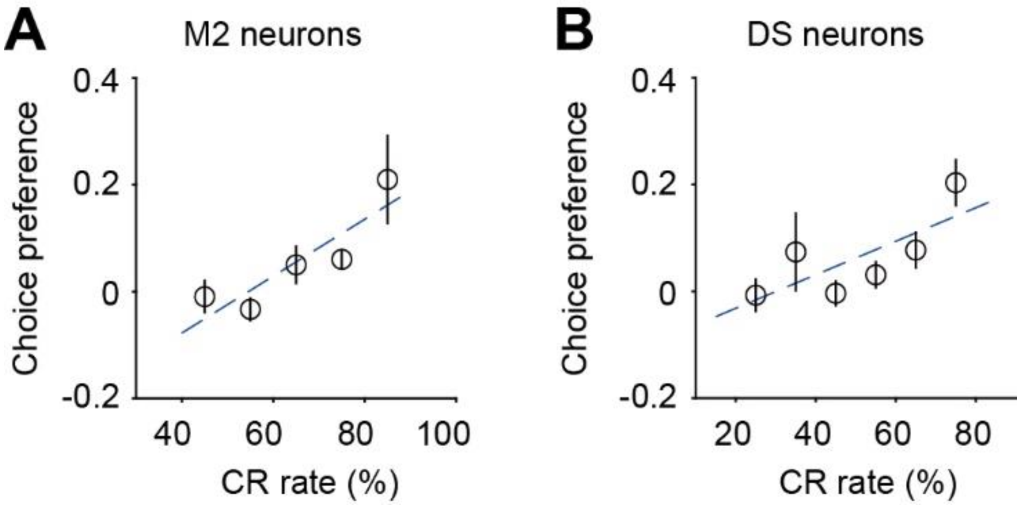

Fig. S6 Choice preference computed using responses in the first 300 ms of the waiting period. **A** Correlation between the choice preference of M2 neurons and CR rate. r = 0.89, P = 0.045. **B** Correlation between the choice preference of DS neurons and CR rate. r = 0.75, P = 0.08. Data are represented by the mean ± SEM.

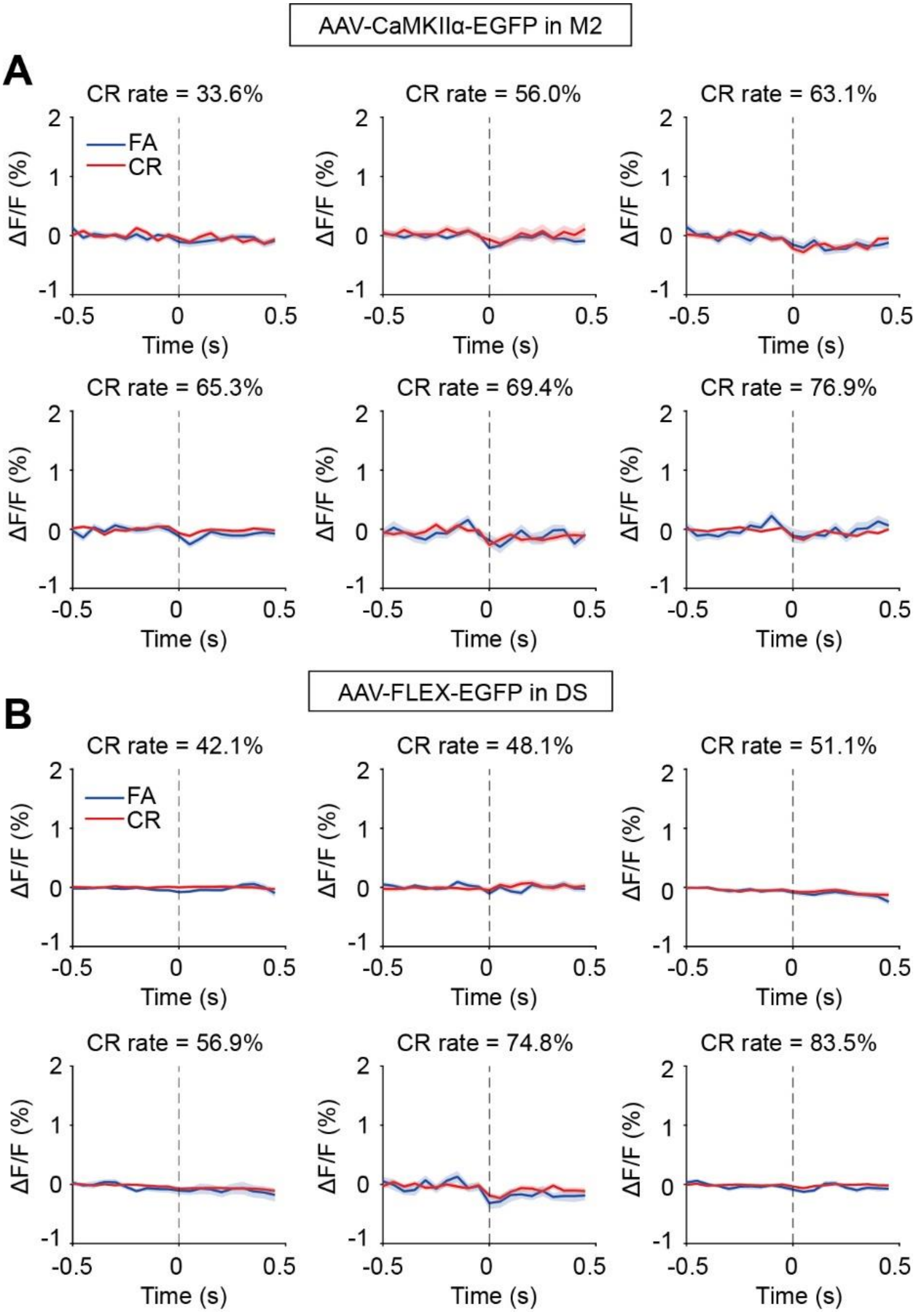

AAV-CaMKIIα-EGFP in M2
A
CR rate = 33.6%
CR rate = 56.0%
CR rate = 63.1%
CR rate = 65.3%
CR rate = 69.4%
CR rate = 76.9%
FA
CR
ΔF/F (%)
Time (s)
AAV-FLEX-EGFP in DS
B
CR rate = 42.1%
CR rate = 48.1%
CR rate = 51.1%
CR rate = 56.9%
CR rate = 74.8%
CR rate = 83.5%
FA
CR
ΔF/F (%)
Time (s)

Fig. S7 Fiber photometry recordings from control mice that expressed GFP in the M2 or the DS. **A** Fiber photometry recording of M2, from 6 sessions of 3 mice in which AAV-CaMKIIα-EGFP was injected into the M2. **B** Fiber photometry recordings of DS, from 6 sessions of D1-Cre mice (n = 2) and D2-Cre mice (n = 2) in which AAV-FLEX-EGFP was injected into the DS. Data are represented by the mean ± SEM.

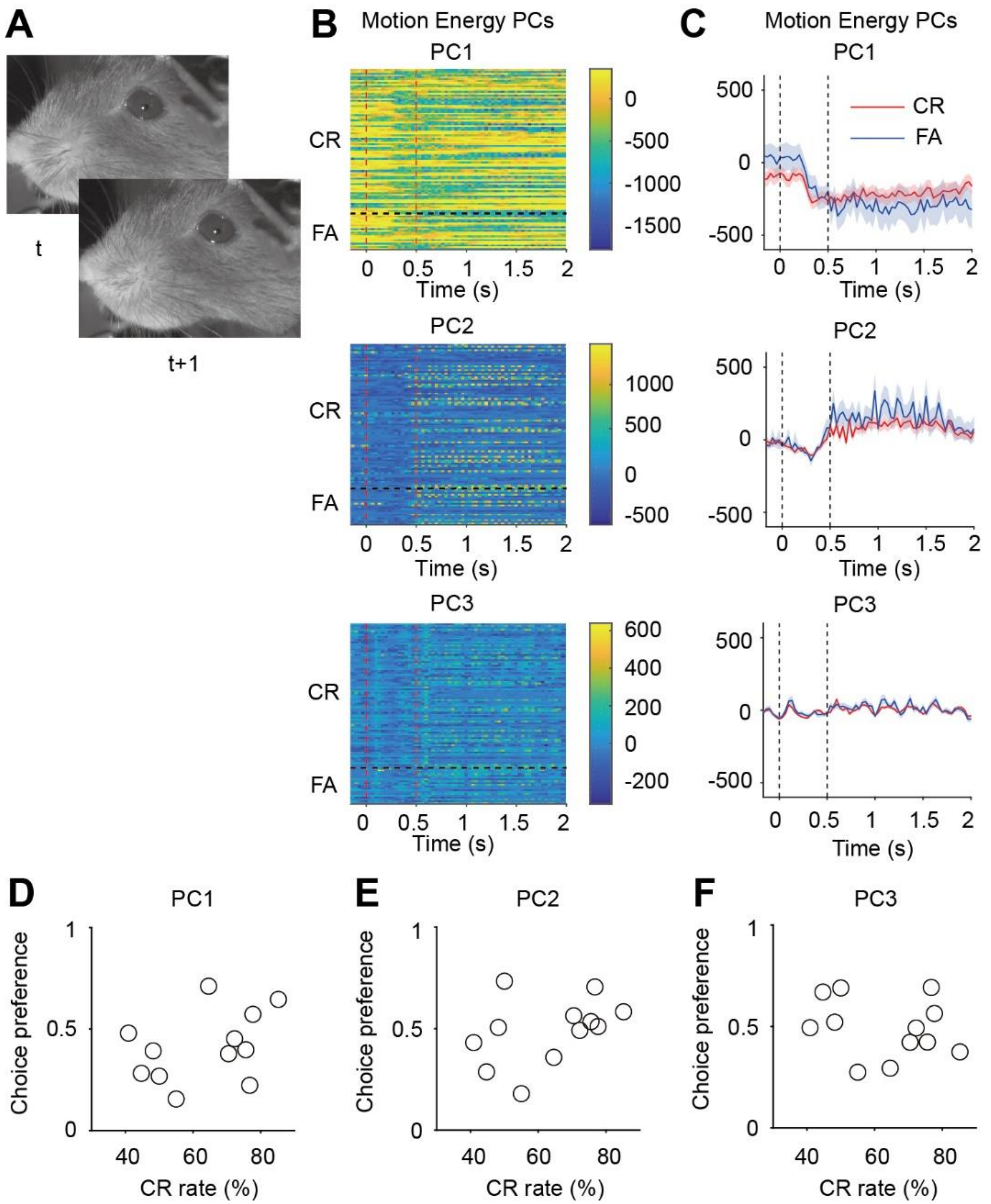

Fig. S8 Analysis of orofacial movements. **A** Two consecutive images from a video recording of a mouse's face during a behavioral task. **B** The top three principal components (PCs) of the motion energy of orofacial movements from an example session. The horizontal dashed line separates CR trials from FA trials. The two vertical red dashed lines indicate the duration of the waiting period. **C**

Motion energy PC averaged over FA trials or CR trials for the example session shown in **B**. The two vertical dashed lines indicate the duration of the waiting period. Data are represented by the mean ± SEM. **D** The choice preference computed using motion energy PC1 in CR trials and FA trials was not significantly correlated with CR rate. $r = 0.37$, $P = 0.23$. **E** The choice preference computed using motion energy PC2 in CR trials and FA trials was not significantly correlated with the CR rate. $r = 0.39$, $P = 0.21$. **F** The choice preference computed using motion energy PC3 in CR trials and FA trials was not significantly correlated with the CR rate. $r = -0.21$, $P = 0.51$. $n = 12$ sessions from 7 mice.

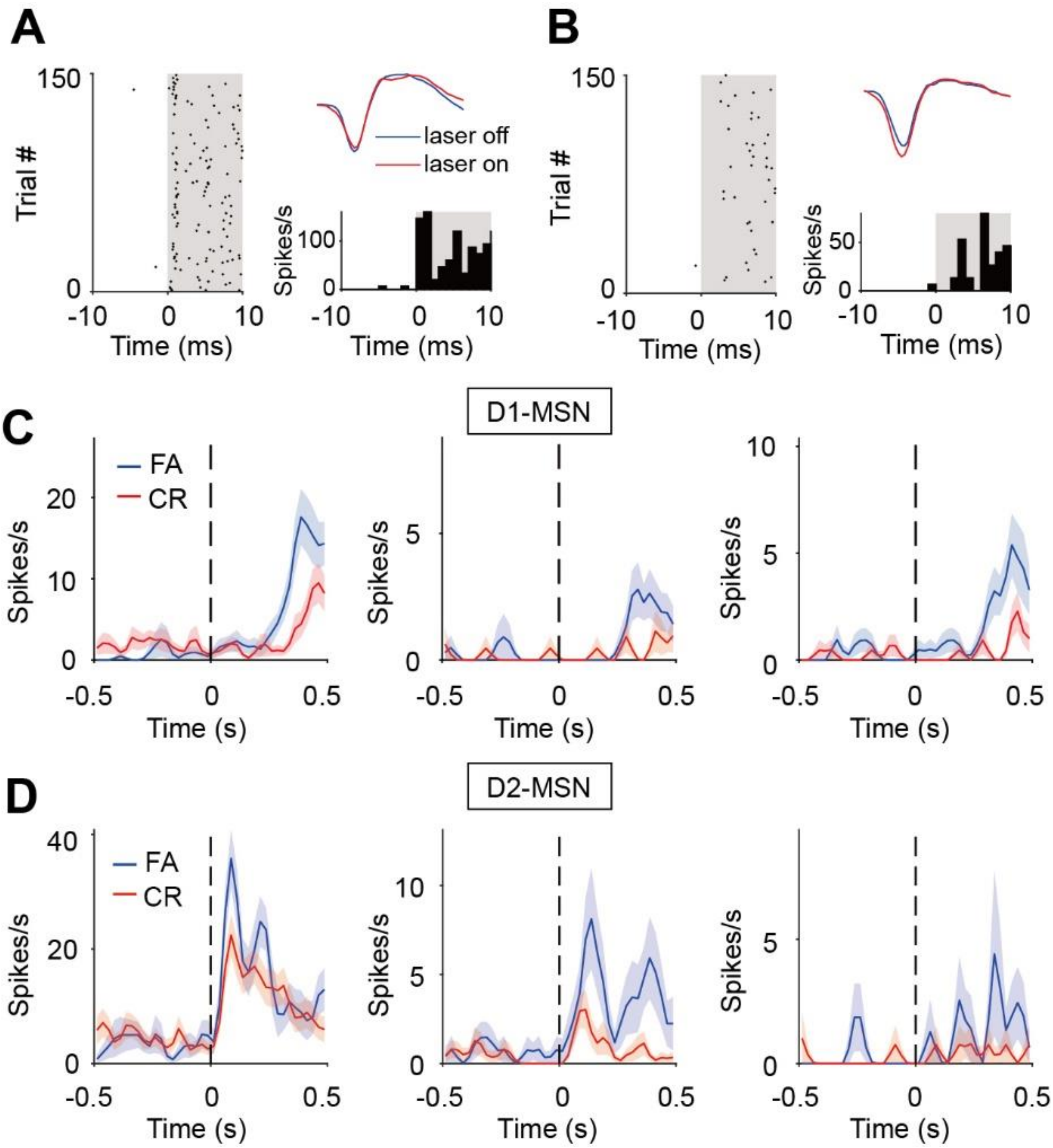

Fig. S9 Choice-related activity in D1-MSNs and D2-MSNs. **A** Spike rasters (left), waveforms, and PSTH (right) of an optogenetically identified D1-MSN. Time 0 is laser onset. Shading, laser stimulation. **B** Spike rasters (left), waveforms, and PSTH (right) of an optogenetically identified D2-MSN. Time 0 is laser onset. Shading, laser stimulation. **C** PSTHs of D1-MSNs (n = 3) in response to No-Go stimulus in FA and CR trials. **D** PSTHs of D2-MSNs (n = 3) in response to No-Go stimulus in FA and CR trials. Data are represented by the mean ± SEM.

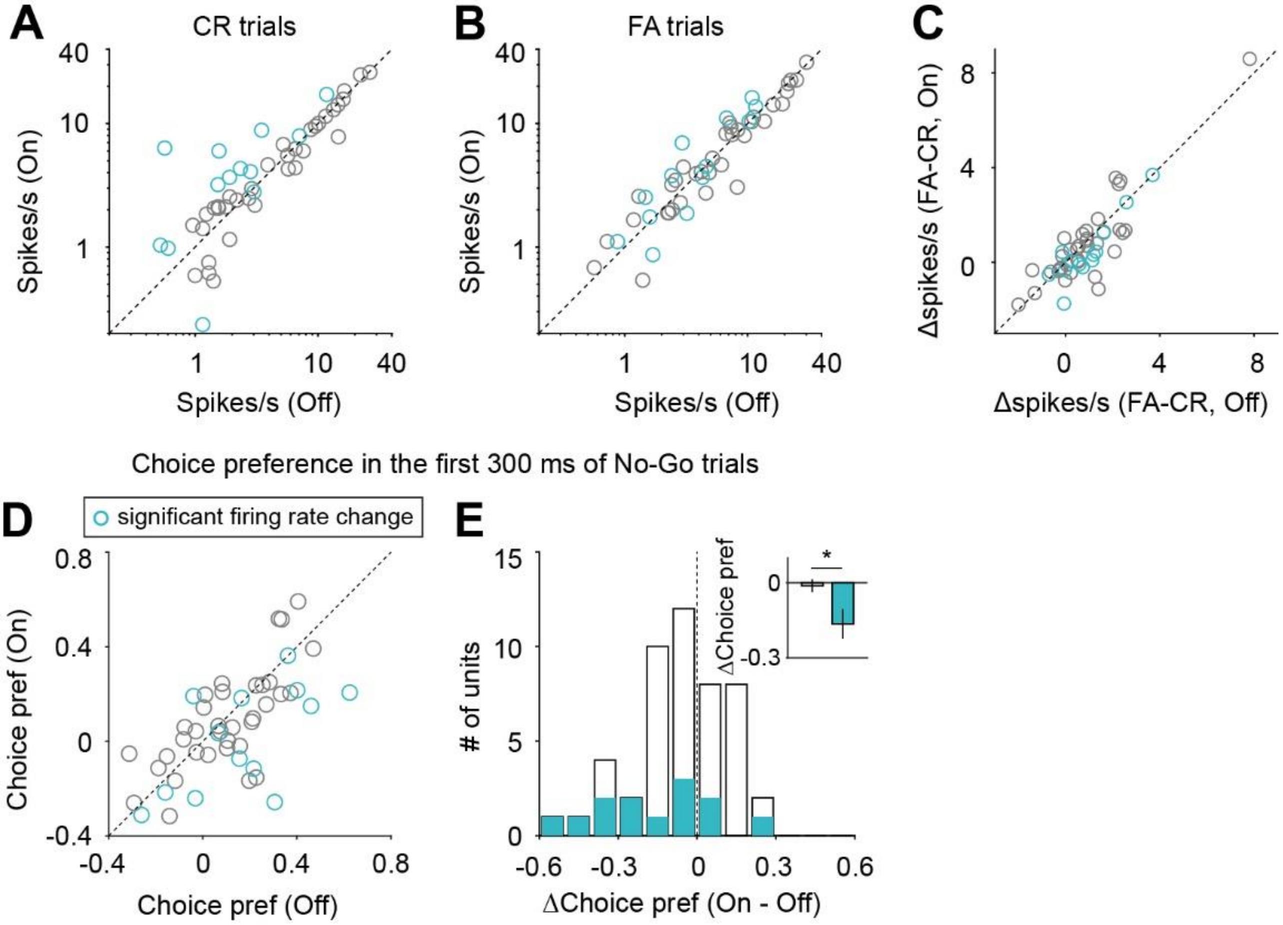

Fig. S10 Effect of inactivating M2DS-p neurons on the firing rates and choice preference of DS neurons. We compared the firing rates of a DS neuron between laser-off and laser-on conditions in CR (FA, or Hit) trials using the Wilcoxon signed rank test, yielding three *P* values. The firing rate was considered to be significantly affected by the inactivation of M2DS-p neurons if one of the three *P* values was < 0.05. **A** Firing rate in laser-off *vs*. laser-on condition for CR trials. $P = 0.17$, $n = 48$ for all DS neurons; $P = 0.003$, $n = 13$ for those DS neurons (blue dots) whose firing rates were significantly affected by the inactivation of M2DS-p neurons. Dashed line, the diagonal line. **B** Firing rates in laser-off *vs.* laser-on condition for FA trials. $P = 0.90$, n = 48 for all DS neurons; $P = 0.08$, $n = 13$ for those DS neurons (blue dots) whose firing rates were significantly affected by the inactivation of M2DS-p neurons. **C** Firing rate difference between FA and CR trials in laser-off *vs.* laser-on condition. $P = 0.15$, $n = 48$ for all DS neurons; $P = 0.01$, $n = 13$ for those DS neurons (blue dots) whose firing rates were significantly affected by the inactivation of M2DS-p neurons. Wilcoxon signed the rank test. **D** Choice preference in the first 300 ms of the waiting period for DS neurons in laser-off *vs.* laser-on trials. $P = 0.097$ and $n = 48$ for all DS neurons, $P = 0.017$ and $n = 13$ for those DS neurons (blue dots) whose firing rates were

significantly affected by the inactivation of $M2_{DS\text{-}p}$ neurons. Wilcoxon signed the rank test. Dashed line, the diagonal line. **E** Distribution of ΔChoice preference (computed using responses in the first 300 ms of the waiting period). Blue, for those DS neurons whose firing rates were significantly affected by the inactivation of $M2_{DS\text{-}p}$ neurons. Inset, comparison of ΔChoice preference between DS neurons with significant firing rate change (blue) and those with insignificant firing rate change (white). * $P < 0.05$, Wilcoxon rank sum test. Wilcoxon rank sum test.

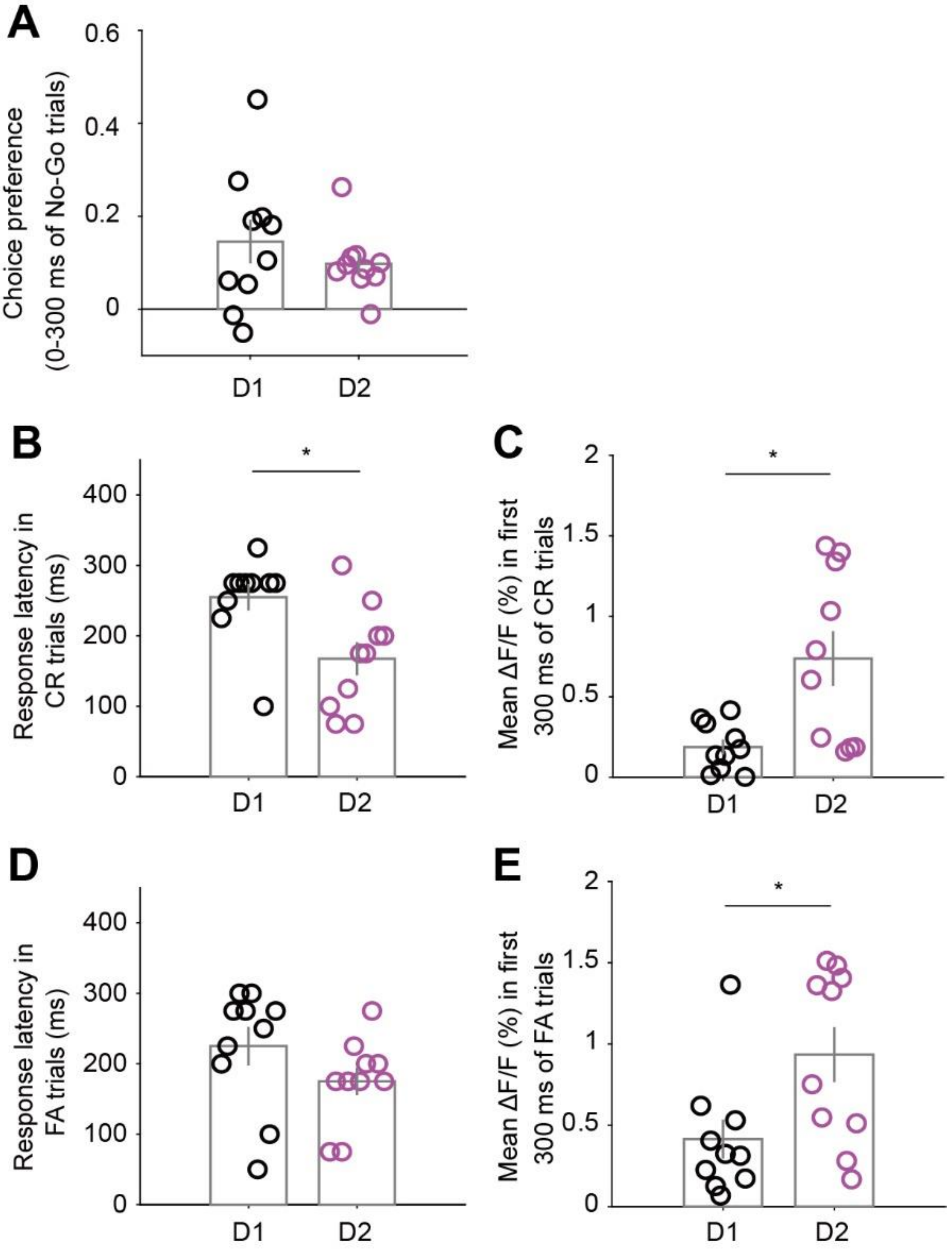

Fig. S11 Comparison of choice preference, response latency and amplitude of early response between D1-MSN$_{M2}$ and D2-MSN$_{M2}$. **A** Choice preference for the responses within the first 300 ms of waiting period was significantly larger than 0 for both D1-MSN$_{M2}$ and D2-MSN$_{M2}$ ($P < 0.01$, Wilcoxon signed

rank test), and the choice preference did not significantly differ between D1-MSN_{M2} and D2-MSN_{M2} ($P = 0.68$, Wilcoxon rank sum test). **B** Response latency of D1-MSN_{M2} and D2-MSN_{M2} in CR trials. $P = 0.013$, Wilcoxon rank sum test. **C** Mean response in the first 300 ms of waiting period in CR trials. $P = 0.011$, Wilcoxon rank sum test. **D** Response latency of D1-MSN_{M2} and D2-MSN_{M2} in FA trials. $P = 0.061$, Wilcoxon rank sum test. **E** Mean response in the first 300 ms of waiting period in FA trials. $P = 0.038$, Wilcoxon rank sum test. $n = 10$ sessions from 5 D1-Cre mice and $n = 10$ sessions from 5 D2-Cre mice. Data are represented by the mean ± SEM.
